# Protocol for the quantification of digestive exophagy in cell culture

**DOI:** 10.1016/j.xpro.2026.104355

**Published:** 2026-03-12

**Authors:** Lucy Funes, Frederick R. Maxfield, Santiago Solé-Domènech

**Affiliations:** 1 Department of Biochemistry and Biophysics, Weill Cornell Medicine, New York, NY 10065, USA

**Keywords:** Cell biology, Cell culture, Cell isolation, Cell-based assays, Microscopy, Neuroscience, Molecular/chemical probes

## Abstract

Microglia use digestive exophagy to partially degrade, extracellularly, Alzheimer’s amyloid-beta aggregates that are too large to be phagocytosed. Here, we present a protocol to quantify this mechanism in cell culture. We describe steps for extracting primary microglial cells and preparing amyloid-beta model aggregates. We then detail procedures for measuring lysosomal exocytosis toward, and extracellular degradation of, these deposits using quantitative fluorescence microscopy. We also provide guidance on quantifying the data using digital image analysis.

For complete details on the use and execution of this protocol, please refer to Jacquet et al.^1^

## Highlights

• Steps for extracting primary murine microglial cells from mouse neonates
• Steps for preparing fluorescently labeled model Alzheimer’s fibrillar Aβ_1-42_ aggregates
• Steps to measure *digestive exophagy* of model Aβ_1-42_ aggregates by microglial cells
• Steps to quantify lysosomal exocytosis and aggregate degradation by image analysis

## Abstract (editor highlights)

Publisher’s note: Undertaking any experimental protocol requires adherence to local institutional guidelines for laboratory safety and ethics.

## Graphical abstract

**Figure:**
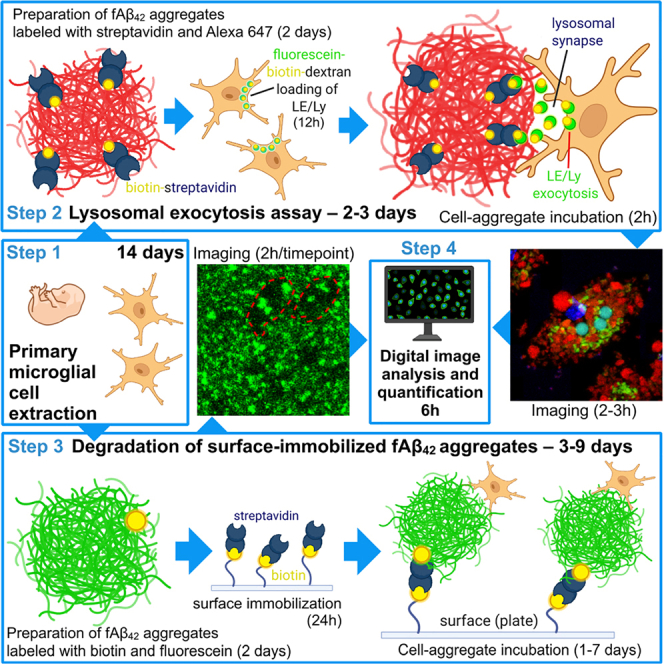

## Before you begin

To digest objects that are too large to be phagocytosed—such as large lipoprotein aggregates similar to those seen in atherosclerotic plaques,^2^^,^^3^ Alzheimer’s disease (AD) amyloid-beta (Aβ) deposits,^1^ or dead adipocytes^4^—phagocytic cells create tightly sealed extracellular regions on the target, into which protons and lysosomal contents are secreted. These compartments, which are called *lysosomal synapses,* are able to degrade extracellular aggregates and some large apoptotic cells.^4^ This process has been called *digestive exophagy*, and it is very similar to the well-known degradative mechanisms used by osteoclasts during bone resorption.^5^ In the AD brain, microglia surround and engage Aβ plaques in an attempt to contain or digest^6^^,^^7^^,^^8^^,^^9^ their diffuse outer layers, which have been described as sources of neurotoxic oligomeric Aβ species.^10^^,^^11^^,^^12^ Digestive exophagy by microglia might help contain Aβ pathology. However, under some circumstances, previously endocytosed lysosomal contents, including fibrillar (f) Aβ, can be released into lysosomal synapses and contribute to plaque growth and/or spread.^1^

Given the novelty and pathological relevance of this mechanism, we prepared two standardized protocols to measure digestive exophagy of fAβ_42_ model aggregates by primary murine microglial cells. Our methodology consists of two sections: measuring lysosomal exocytosis toward lysosomal synapses established on fAβ_42_ aggregates, and measuring the extracellular degradation of surface-immobilized fAβ_42_ fibrils. The combination of these two readouts provides a direct assessment of digestive exophagy. We also developed protocols to image and quantify the pH of lysosomal synapses created by microglial cells on fAβ_42_ model aggregates. For these assays we typically use fluorescein or our new pH sensor ApHID^13^ as pH-sensitive reporters. For more details on these procedures, please refer to Jacquet et al.^1^ and Solé-Domènech et al.^13^

For experiments described here, we used primary murine microglial cells, and we provide our optimized extraction protocol. Nevertheless, other types of phagocytes such as primary macrophages can be used instead. For all assays, we will prepare model fAβ_42_ aggregates labeled with fluorescein, Alexa 647, biotin and/or streptavidin. For the lysosomal exocytosis assay, we will load microglial late endosomes and lysosomes (LE/Lys) with 10 KDa dextran polymers labeled with fluorescein, and measure dextran exocytosis towards model aggregates. Dextrans have been established lysosomal tracers for >40 years (reviewed in Solé-Domènech et al.^13^) and used in parallel with other organelle markers such as LAMP1. In our assay, 10 kDa dextrans are internalized and chased to ensure localization LE/Lys. Hence, exocytosed signal necessarily derives from these compartments. Furthermore, to measure the extracellular degradation of fAβ_42_ aggregates under conditions that impede their endocytosis or phagocytosis, we will break them down into smaller fragments using sonication and immobilize them on glass-like polymer surfaces using biotin-streptavidin chemistry. Lastly, for these experiments, we report digital image quantification approaches using Metamorph software. However, clear steps are provided in order to carry out image analysis using any available image quantification tool.

### Innovation

The protocol we present is the *first standardized approach* for quantifying digestive exophagy of Alzheimer’s-like fAβ_42_ aggregates in cell culture. We do this by describing two fundamental measurements, namely the exocytosis of lysosomal contents toward model fAβ_42_ aggregates, and the degradation of surface-immobilized aggregates, by microglial cells in cell culture. These constitute two fundamental readouts in the assessment of this mechanism in cell culture. Our workflow is not equivalent to previously described aggregate clearance assays, and represents a distinct mechanism of microglial degradation which occurs *extracellularly*. The focal study to which this protocol refers to demonstrates digestive exophagy of model fAβ_42_ aggregates by microglial cells in cell culture, and the mechanism was validated ex vivo in 5xFAD mouse brain tissue sections by visualization of acid phosphatase staining using electron microscopy.^1^ Additionally, we also described digestive exophagy of large lipoprotein aggregates similar to those seen in atherosclerotic plaques^2^^,^^3^^,^^14^ or dead adipocytes^4^ by macrophages. Outside of our work, digestive exophagy has been confirmed in amoebae digesting bacterial biofilms.^15^ Also, acidified compartments similar to lysosomal synapses have been observed during osteoclast bone resorption using intravital imaging microscopy.^16^ For complete details on the established novelty of digestive exophagy, please refer to Jacquet et al.^1^

### Institutional permissions

All experiments involving animals were carried in accordance with ethical protocols reviewed and approved by our Institutional Animal Care and Use Committee. Undertaking any experimental protocol involving use of animals for experimentation requires adherence to local institutional guidelines for laboratory safety and ethics.

### Primary murine microglial cell extraction

**Timing: 14 days**

1. Prepare a sterile cell culture hood or a ductless ventilation hood.
  a. Sanitize working area with 70% ethanol and expose to ultra-violet light for 20 min prior to use.
  b. Bring in a stereotaxic microscope equipped with a front lamp unit, or place a separate light source next to the microscope.
    ***Note:*** Proper illumination during the procedure is important. You will be working with very small tissue parts.
  c. Prepare the following tools:
    Operating scissors, curved tip forceps, fine tip forceps, micro-spatula, fine tip tweezers and scalpel.
  d. Sterilize tools by autoclave.
  e. Immediately prior to use, submerge tools in a small beaker filled with 70% ethanol with paper wipes covering the bottom.
  f. Bring tools inside your sterile workstation.
  g. Spray a Styrofoam box and a few small ice packs with 70% ethanol.
  h. Place the ice packs inside the box and bring box inside your sterile workstation.
  i. Bring three 50 mL Falcon tubes filled with CMF-PBS and a 10 cm sterile Petri dish to the box.
  j. Place the Petri dish on top of the ice packs.
  k. Fill the Petri dish with cold CMF-PBS and keep refrigerated on top of ice packs.
  l. Place a second, sterile Petri dish under the stereotaxic microscope.
    ***Note:*** This will be your working area during brain extraction and manipulation.
  m. Open and place a small biohazard bag near the work area for fast disposal of dissected mouse pups.
2. Select mouse neonates aged 2–3 days old from your breeding cages.
  a. Transport neonates to your workstation using an approved container.
  b. Sterilize the outside of the container with 70% ethanol.
  c. Place container inside your sterile work area.
3. Collect pup heads.
  a. Place a pup over the Petri dish.
  b. Wipe the top of its head with a paper wipe soaked in 70% ethanol.
  c. Euthanize the animal by decapitation using the sterilized scissors.
  d. Dispose the carcass in a biohazard bag, and keep the head.

***Note:*** Pups should be euthanized, and brains collected, one at a time.

4. Extract the brain:
  a. Using sterilized tweezers with your non-dominant hand, anchor the head by the nose area.
  b. With your dominant hand, use a sterilized scalpel to make an incision from the front of the head to the back.
    ***Note:*** Cut only the skin layer.
  c. Use tweezers to move skin to the side of the incision.
    ***Note:*** To do this, hold the tweezers with your non-dominant hand and hold the head by its sides, gently pulling the skin downwards in order to open the incision site.
  d. Repeat the incision, this time cutting through the skull, being careful not to puncture the brain.
  e. Continue holding the head with the tweezers and pull downwards as described above in order to move the opened skull to the sides.
    ***Note:*** Allow for sufficient space to insert the sterilized spatula by gently sliding it under the brain on a sideways motion. Once inserted, bring the spatula upwards and carry the brain with it in a gentle “scooping” motion.
  f. Place the extracted brain on the Petri dish filled with cold CMF-PBS.
5. Repeat step 3 above for all pups, Leave dissected brains aside, in cold CMF-PBS.

***Note:*** The scalpel will become dull after processing 3–4 brains. Switch to a new scalpel.

**Pause point:** Take a break before proceeding to removing the meninges.

6. Remove the meninges from all brains:
  a. Wipe down a fresh Petri dish with 70% ethanol and add cold CMF-PBS to the midway mark.
  b. Shorten the tip of a plastic transfer pipette by cutting it with scissors.
  c. Use the pipette to carefully pick up and transfer brains from inside the Petri dish to your work area.
    ***Note:*** Cutting off the small terminal tip of the pipette reduces the risk of damaging the brains during transfer.
  d. Transfer one brain to a fresh Petri dish placed under the microscope and add more CMF-PBS to allow the brain to float.
  e. Using dull forceps in the non-dominant hand, hold the brain by clamping it down.
    ***Note:*** Clamp it down by the olfactory bulb or cerebellum and maintain the brain held in that position.
  f. While anchoring the brain with the non-dominant hand, use sharp forceps in the dominant hand to carefully remove the meninges from the entire surface area of the cortex first.
  g. Remove the membrane from ventral regions next.
    ***Note:*** Removing the meninges will look like unwrapping a plastic cover from an object. The membrane might break into pieces. Try to grab onto protruding bits of the membrane and gently pull them in order to carry as much membrane as possible.
7. Collect the clean cortical areas:
  a. Use sharp tweezers in the dominant hand to cut away the right and left cortical areas from the rest of the brain.
    ***Note:*** This will resemble pulling the cap of a mushroom from its stem.
  b. Use the plastic transfer pipette to pick up the cortical areas and transfer them to a 50 mL centrifuge conical tube filled with 2 mL of cold CMF-PBS.
    ***Note:*** Keep the cortical areas in cold CMF-PBS at all times.
  c. Use the transfer pipette to discard all remaining brain tissue into the biohazard bag.
8. Repeat steps 5–6 above for all brains.
9. Allow cortices to settle in the buffer until they sit at the bottom of the tube.

***Note:*** Do not collect cortices from more than 4-5 mice in a single centrifuge conical tube.

10. Warm up complete DMEM medium, trypsin, and DNAse aliquots at 37 °C in a water bath.

***Note:*** Do not keep trypsin and DNAse in the water bath for more than 30 min. As medium and reagents warm up, clean up your workstation and make space for the next steps.

11. Homogenize cortices in CMF-PBS:
  a. Carefully aspirate the top layer of your CMF-PBS buffer in each conical tube and wash 3X with 3 mL of fresh buffer.
    ***Note:*** Be careful not to aspirate any cortices.
  b. After the last wash, add 5 mL of fresh CMF-PBS buffer, and mince the cortical tissues.
    ***Note:*** Use a scalpel and triturate about 30 times. Allow the minced tissues to settle.
12. Trypsinize cortices:
  a. Remove the top layer of your CMF-PBS buffer.
  b. Add 1 mL of warm trypsin solution containing trypsin, DNAse and MgSO_4_ using a 5 mL serological pipette.
  c. Pipette up and down 3 times.
  d. Incubate the trypsinization mixture in a water bath at 37 °C for 5 min.

**CRITICAL:** During incubation, swirl the tube gently once per minute.

13. Homogenize the cortices:
  a. After 5 min incubation in trypsin, bring conical tubes back to your workstation.
    ***Note:*** At this point, the cortical areas should have turned into a gelatinous substance.
  b. Place a rubber bulb at the end of a glass pipette and use it to carefully pick up the cortices.
  c. Transfer the gelatinous tissue to a 15 mL conical tube containing 2 mL of warmed DNAse buffer.
    ***Note:*** Have one 15 mL conical tube filled with DNAse for each 50 mL conical tube containing trypsinization mix ready.
  d. Use the glass pipette with attached rubber bulb to pipette your mixture up and down until cortices are fully homogenized.
  e. Spin the mixture at 400 × *g*. for 5 min.
14. Collect and plate the mixed cell culture:
  a. After centrifugation, aspirate the top layer of DNAse carefully and discard.
    ***Note:*** There will be a pellet left over at the bottom of the 15 mL tube, separated into two layers. The top layer is lighter in color and contains cells, while the bottom layer should appear reddish and contains residual debris and meninges.
  b. Using a fresh glass pipette with attached rubber bulb, very carefully collect the top, light colored, pellet layer and transfer to a 50 mL conical tube containing 10 mL of DMEM medium.
  c. Resuspend the pellets by gently pipetting up and down a few times. Transfer each pellet to a separate tube.
    **CRITICAL:** Avoid collecting the bottom, reddish layer of the pellet as this will interfere with the cell culture and cause toxicity.
  d. Add an additional 10 mL of DMEM to each new conical tube and transfer into a 75 cm^2^ air vented T75 tissue culture flask.
  e. Label the flask with the date, # of brains contained in the pellet, age of pups and any other relevant information.
    **CRITICAL:** Each pellet in a given conical tube (containing cortices from 4-5 mice) should be transferred to a separate flask. Do not transfer more tissue than that into a flask, as this will interfere with growth and viability.
15. Place flasks in the incubator at 37 °C with 5% CO_2_.
16. After a 2-day incubation period at 37 °C, replace the medium in each culture flask:
  a. Collect the medium in each flask and transfer it to a separate 50 mL conical tube.
  b. Add 10 mL of fresh media in each flask immediately after.
    **CRITICAL:** Do not leave the flask without media, as cells will dry up.
  c. Spin down the collected media at 500 × *g*. for 4 min.
  d. Once done spinning, filter the medium through a 0.4-micron filter to remove debris.
  e. Rewarm the filtered media to 37 °C in the incubator.
  f. Wash the flasks by gently rinsing with 10 mL of warm DMEM.
  g. Repeat the wash 3X.
  h. Discard the medium.
  i. Add back 15 mL of filtered medium to each flask and add 10 mL of fresh medium.
  j. Return flasks to the incubator.
17. 10–14 days after microglial cell extraction, cell cultures should be ready to harvest.

**Figure 1:**
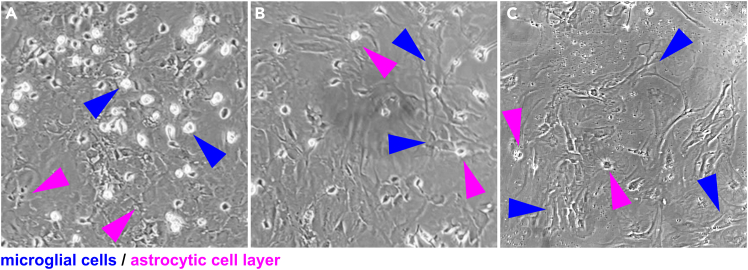
Astrocytic-microglial cell co-cultures in 75 cm^2^ tissue-treated culture flasks Astrocytic cells form a layer (purple arrowheads) sitting underneath microglial cells, which appear as small bright dots under the light microscope (blue arrowheads). Mixed cell cultures were visualized on a 75 cm^2^ culture flask using a light microscope. Some areas in the flasks show varying degrees of microglial cell densities (A, B). This usually correlates with how confluent the astrocytic cell layer sitting underneath is. Sparse or sub-optimal astrocytic cell layers will generally lead to very low microglial cell densities (C).

**CRITICAL:** When inspecting the flasks under the microscope, an astrocytic cell layer (flat, elongated cells with dark contrast, Figures 1A and 1B, purple arrowheads) must have grown underneath the microglial cells, which will be sitting on top and appear as small, bright dots (Figures 1A and 1B, blue arrowheads).

**Figure 2:**
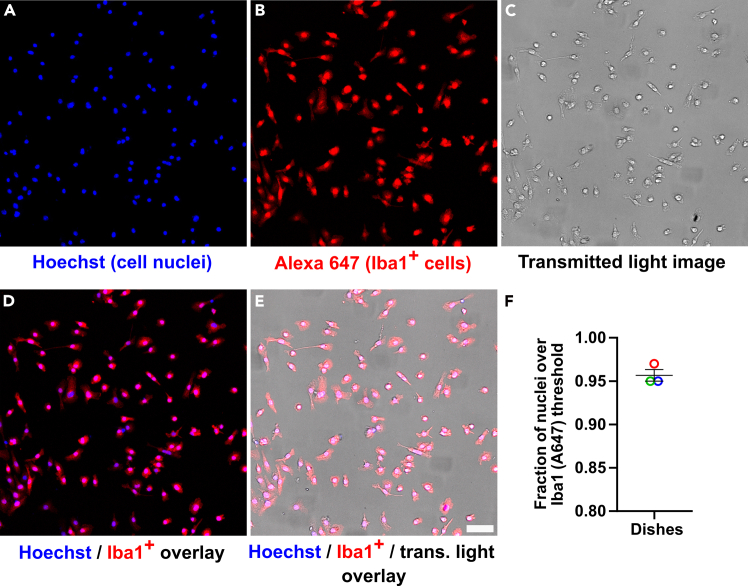
Microglial cell culture purity verified by Iba1 immunostaining (A–E) Microglial cells extracted following the protocol described above were seeded on poly-d-lysine-coated coverslips attached to 35-mm dishes at 20,000 cells per chamber, fixed in 4% PFA for 15 min and stained with the nuclei marker Hoechst and the microglial cell-specific antibody Iba1 followed by confocal imaging. Cell nuclei (A), Iba1 staining detected with the secondary antibody Alexa 647 (B) and transmitted light channel (C), together with overlays (D and E) are shown. Iba1 immunostaining (Alexa 647) colocalized with each cell nuclei (Hoechst) was quantified. Cell nuclei with associated Alexa 647 average intensities above 800 grayscales (way above background fluorescence signal) were classified as Iba^+^ and quantified accordingly. Iba^+^-to-Hoechst cell ratios were calculated and plotted for each dish imaged (F, geometric shapes, 3 in total). We obtained an average microglial cell culture purity of 96% (0.96 ± 0.01). Bars indicate averaged ratio ±SD. All images were corrected for background fluorescence by subtracting the 5^th^ intensity percentile. Scale bar: 50 μm.

18. Extract microglia from the flasks:
  **CRITICAL:** For best results, cells should be used 10–14 days post extraction. Flasks older than 14 days should be carefully inspected before harvesting cells. Often, cells kept for too long in the flask will accumulate various debris resembling membrane fragments and vesicular structures that appear bright under the light microscope. If this is observed, harvest cells within 24 h or discard altogether.
  a. Aspirate medium from flasks and replace with 20 mL of fresh, warm DMEM without phenol red.
  b. Return flasks to the incubator and allow cells to settle in the fresh media for 1 h at 37 °C.
  c. Prepare a heater blower and an orbital shaker. Spray down surfaces and tools with 70% ethanol.
  d. Take the flasks out of the incubator and wrap parafilm around their caps in order to maintain the pH of the medium.
    **CRITICAL:** Make sure the flask is tightly sealed with paraffin in order to prevent alkalinization of the cell medium.
  e. Place flasks on the orbital shaker, adjust the heater blower to 37 °C and turn it on.
  f. Turn on the shaker and let the flasks shake for 2 h at 1–3 cycles per second.
    ***Note:*** Direct the warm air toward the flasks so that the medium is maintained warm, but do not bring the flasks too close to the heater. Microglial cells will gently lift during shaking and accumulate in the media.
  g. Following shaking, transfer your flasks to your sterile workstation and collect the medium with cells in 15 mL centrifuge conical tubes (10 mL per tube).
  h. Add 20 mL of fresh medium to each empty flask and rinse to dislodge any microglia still loosely adhered to the flask.
    **CRITICAL:** Do not rinse too vigorously. Doing so may lift the astrocytic cell layer underneath microglial cells.
    ***Alternatives:*** to increase microglial cell harvesting, the astrocytic cell layer can be removed by mild trypsinization. This will free up additional microglial cells sitting underneath. For complete details on this procedure, please refer to Saura et al.^17^
  i. Collect the medium containing the resuspended cells.
  j. Transfer it to the 15 mL centrifuge conical tubes.
  k. Spin all conical tubes containing the microglial cell resuspension at 500 × *g*. for 4 min.
    ***Note:*** After spinning, a pellet will be visible at or near the bottom of the tube. The pellet will appear as a small brownish dot, and a marker should be used to mark its location in order to avoid accidental aspiration during washing steps.
  l. Aspirate the supernatant from all conical tubes.
  m. Resuspend all pellets in a total of 1 mL of fresh, warmed DMEM medium without phenol red.
  n. Count cell density using a cell counter.
  o. Prepare a working stock in warm DMEM without phenol red as needed.
  p. Seed microglial cells.
    ***Note:*** Our microglia extraction protocol results from modifications of previous methodologies published by our lab and others. These protocols typically yield microglial cell population purities of ∼90%.^18^^,^^19^^,^^20^ Seeded microglia will present amoeboid morphology homogeneously distributed over the seeding surface. If necessary, to verify purity, we recommend co-staining with Hoechst (for total cell count) and Iba1 (specific microglial cell marker)^19^ as described by Stark et al.^21^ We show a representative staining of our extracted microglial cells seeded in poly-d-lysine -coated dishes below (Figure 2).

### Preparation of fAβ_42_ aggregates labeled with Alexa 647 and streptavidin for the lysosomal exocytosis assay

**Timing: 2 days**

19. Label monomeric Aβ_42_ peptide with Alexa 647-NHS ester at Aβ_42_: Alexa 647 molar ratio of 1:4, according to the following recipe:

**Table:** Monomeric Alexa 647-Aβ_42_ peptides

Reagent	Stock concentration	Amount	Final concentration
Aβ_42_ monomeric, unlabeled	1 mg/mL in sodium tetraborate	1000 μL	221 μM
Alexa 647-NHS (MW: 1025.2 g/mol) (1 mg vial in 100 μL DMSO)	10 mg/mL in DMSO (9754 μM)	100 μL	886 μM
**Total**	–	**1100 μL**	**∼200 μM Aβ**_**42**_

**Storage conditions:** −80 °C. Use within six months.

  **CRITICAL:** We purchased all monomeric Aβ_42_ peptides from the vendor Anaspec. The company analyzes their Aβ_42_ peptides by HPLC and mass spectrometry to verify purity and makes the results available to their customers. Aβ_42_ can be purchased from other vendors, but we highly recommend requesting purity analysis results first.
  **CRITICAL:** When working with Aβ_42_ peptides, all solutions and reagents should be kept cold in order to prevent peptide aggregation during handling.
  a. Solubilize 1 mg of monomeric Aβ_42_ peptide in 1000 μL of cold 50 mM sodium tetraborate pH 9.3 buffer. Keep refrigerated in ice.
  b. Prepare a stock of 10 mg/mL solution of Alexa 647 NHS ester in dry DMSO.
    ***Note:*** To do that, solubilize 1 mg of NHS ester in 100 μL of DMSO.
  c. Transfer the Alexa 647 NHS ester stock solution to the unlabeled Aβ_42_ monomeric peptide solution.
  d. Incubate the mixture for 1h at 4°C with constant, slow rotation.
20. Following reaction, dialyze the reaction mixture to discard unreacted Alexa 647 (hydrolyzed ester).
  a. Fill a sterile beaker with 4 L of sterile, cold 50 mM sodium tetraborate pH 9.3 buffer. Add a magnetic stir bar.
  b. Load the Aβ_42_ crude labeling reaction into a 3.5 KDa cut-off dialysis cassette.
  c. Attach the cassette to a sterile foam holder and transfer to the beaker containing sodium tetraborate.
  d. Cover with aluminum foil. Place beaker on a magnetic stirrer and stir for 3 h at 4 °C.
  e. After 3 h, replace dialysis buffer with fresh sodium tetraborate and dialyze for 12h. Next morning, complete a third, 3h dialysis round.
21. Extract the dialyzed Alexa 647-Aβ_42_ peptide from the dialysis cassette using a sterile 1 mL syringe and split into 50 μL aliquots in microcentrifuge tubes. Store at – 80 °C.
22. Determine Alexa 647 incorporation by absorbance.

***Note:*** Alexa 647 incorporation into soluble Aβ_42_ peptide must be determined by absorbance. This will allow for calculation of how much Alexa 647-Aβ_42_ peptide to add to the Aβ aggregation mixture to achieve a specific Aβ:Alexa 647 ratio (see calculations below). The batch used in our experiments was labeled at an Aβ_42_ : Alexa 647 molar ratio of 1:1.12 (221 μM Aβ_42_ and 249 μM Alexa 647).

23. Prepare a fAβ_42_ aggregation mixture by adding Alexa 647-Aβ_42_, biotin-Aβ_42_ and unlabeled Aβ_42_ monomeric peptides together at 1:0.05:0.17 molar ratios according to the following recipe:

**Table:** Alexa 647-biotin-Aβ_42_ aggregates

Reagent	Stock concentration	Amount	Final concentration
Aβ_42_ monomeric unlabeled	7.14 mg/mL peptide (1 mg solubilized in 123 μL sodium tetraborate)	123 μL	157 μM
Aβ_42_–A647 monomeric (custom label)	1.0 mg/mL Aβ_42_ peptide in sodium tetraborate, labeled with Alexa 647 at an Aβ_42_:A647 molar ratio of 1:1.12 (249 μM A647∗, 221 μM Aβ_42_ in peptide)	49.5 μL	8.88 μM Aβ_42_ 10 μM A 647
Aβ_42_–Biotin monomeric (commercially available)	1.75 mg/mL Aβ_42_-biotin peptide (0.2 mg solubilized in 114 μL sodium tetraborate). Peptide commercially available labeled at Aβ_42_: biotin of 1:1 ratio.	114 μL	33.3 μM Aβ_42_ 33.3 μM biotin
PBS	1 X	945 μL	∼0.74 X
HCl	1 N	40 μL	∼0.03 N
**Total**	–	**1272 μL**	**∼200 μM Aβ**_**42**_

∗Determined by absorbance. Abbreviations: A647: Alexa Fluor 647.
  **Storage conditions:** 4 °C. Use within a month. Do not freeze.
  a. Solubilize 1 mg of monomeric Aβ_42_ peptide in 123 μL of cold 50 mM sodium tetraborate pH 9.3 buffer. Keep refrigerated in ice.
  b. Solubilize 0.2 mg of monomeric Aβ_42_-biotin peptide in 114 μL of cold 50 mM sodium tetraborate pH 9.3 buffer. Keep refrigerated in ice.
  c. Add the various monomeric Aβ_42_ components together for fibrillation (see table above) into a 1.5 mL tube and complete volume with 1X PBS. Vortex briefly.
  d. Add 40 μL of a 1N HCl solution and measure pH using pH-indicator strips.
  e. If necessary, adjust the pH of the solution by adding small volumes of the 1N HCl solution.
    ***Note:*** Add 1N HCl until the pH indicator strip color matches that of pH of ∼5 in the pH color scale.
    **CRITICAL:** If a pH lower than 5 is reached, proceed to the fibrillation step anyway. Do not correct the acidity of the solution with NaOH. We have observed a dramatically reduced fibrillation yield when NaOH is added to a fibrillation mixture.
  f. Incubate for 12-18h with constant rotation at 37 °C in a convection oven.
  g. After incubation, centrifuge the mix at 21,000 × *g*. for 10 min.
  h. Carefully remove the fluorescent supernatant without disturbing the pellet.
  i. Resuspend pellet in 1 mL of fresh 1X PBS and spin again.
    **CRITICAL:** Repeat step f above until the supernatant appears clear. The reaction mixture will contain unreacted soluble Aβ_42_ peptides that need to be completely eliminated by several centrifugation steps prior to addition of streptavidin. This will ensure that only fibrillar Aβ_42_ species (and not soluble ones) are being linked to streptavidin.
24. Label fAβ_42_-biotin-Alexa 647 aggregates with streptavidin.
  a. Prepare a 1 mM stock solution of streptavidin (54.3 mg in 1 mL of 1X PBS).
  b. Carefully remove the supernatant from the fAβ_42_-biotin-Alexa647 pellet.
  c. Add the totality of your 1 mM streptavidin solution. Resuspend the pellet and incubate at 37 °C under constant rotation for 1 h.
  d. After incubation, spin the mixture at 21,000 × *g*. for 10 min and carefully remove the supernatant. Repeat 3X.
    **CRITICAL:** Unreacted streptavidin must be completely removed from the aggregates in order to avoid interference during the lysosomal exocytosis assay.
  e. Finally, resuspend the pellet in 1.5 mL of 1X PBS (final 170 μM fAβ_42_ species in solution) and store.
    ***Note:*** Store at 4 °C (for use within 1 to 2 weeks) or at −80 °C until used for experiments.

### Preparation of small fAβ_42_ aggregates labeled with fluorescein and biotin for surface immobilization

**Timing: 2 days**

25. Label monomeric Aβ_42_ peptide with fluorescein-NHS at Aβ_42_:fluorescein molar ratio of 1:4, according to the following recipe:

**Table:** Monomeric fluorescein-Aβ_42_ peptides

Reagent	Stock concentration	Amount	Final concentration
Aβ_42_ monomeric unlabeled	1 mg/mL in sodium tetraborate	1000 μL	221 μM
5/6-carboxyfluorescein-NHS) (MW: 473.4 g/mol) (1 gr vial in 20 mL DMSO)	50 mg/mL in DMSO (106×10^6^ μM)	8.4 μL	886 μM
**Total**	–	**∼1007 μL**	∼221 μM Aβ_42_
  **Storage conditions:** −80 °C. Use within six months.
  a. Solubilize 1 mg of monomeric Aβ_42_ peptide into 1000 μL of cold 50 mM sodium tetraborate pH 9.3 buffer. Keep refrigerated in ice.
  b. Prepare a stock of 50 mg/mL solution of fluorescein-NHS ester in dry DMSO.
    ***Note:*** To do that, solubilize 1 gr of fluorescein NHS ester in 20 mL DMSO, (prepare 0.5 mL aliquots and freeze the ones you won’t be needing).
  c. Add 8.4 μL of fluorescein-NHS stock solution to the unlabeled monomeric Aβ_42_ peptide solution.
  d. Incubate for 1h at 4°C with constant, slow rotation.
26. Dialyze the crude reaction into a 3.5 KDa cut-off dialysis cassette as described in ‘*Preparation of Aβ*_*42*_ *aggregates labeled with Alexa 647 and streptavidin*’ section above.
27. Extract dialyzed, labeled peptide from the dialysis cassette. Split into 50 μL aliquots in microcentrifuge tubes and store at – 80 °C.
28. Determine fluorescein incorporation into Aβ_42_ peptide by absorbance.

***Note:*** The batch used in our experiments was labeled at an Aβ_1-42_:fluorescein molar ratio of 1:0.93 (221 μM Aβ_42_ and 206.16 μM fluorescein).

29. Prepare an aggregation mixture containing soluble fluorescein-Aβ_42_, biotin-Aβ_42_ and unlabeled Aβ_42_ peptides at Aβ_42_:fluorescein and 1:0.05:0.17 molar ratios according to the following recipe:

**Table:** Fluorescein-biotin-Aβ_42_ aggregates

Reagent	Stock concentration	Amount	Final concentration
Aβ_42_ monomeric unlabeled	7.14 mg/mL peptide in sodium tetraborate	122 μL	156 μM
Aβ_42_–fluorescein monomeric (custom label)	1.0 mg/mL Aβ_42_ peptide in sodium tetraborate, labeled with fluorescein at an Aβ_42_:fluorescein molar ratio of 1:0.93 (206 μM fluorescein∗, 221 μM Aβ_42_ in peptide)	60 μL	10.7 μM Aβ_42_ 10 μM fluorescein
Aβ_42_–Biotin monomeric (commercially available)	1.75 mg/mL Aβ_42_ peptide in sodium tetraborate, labeled with biotin at an Aβ_42_: biotin molar ratio of 1:1.	114 μL	33.3 μM Aβ_42_ 33.3 μM biotin
PBS	1 X	936 μL	∼0.74 X
HCl	1 N	40 μL	∼0.03 N
**Total**	–	**1272 μL**	**∼200 μM Aβ**_**42**_

∗Determined by absorbance.
  **Storage conditions:** 4 °C. Use within a month. Do not freeze.
  a. Solubilize 1 mg of monomeric Aβ_42_ peptide in 123 μL of cold 50 mM sodium tetraborate pH 9.3 buffer. Keep refrigerated in ice.
  b. Solubilize 0.2 mg of monomeric Aβ42-biotin peptide in 114 μL of cold 50 mM sodium tetraborate pH 9.3 buffer. Keep refrigerated in ice.
  c. Add the various Aβ42 components together for fibrillation (see table above) into a 1.5 mL tube and complete volume with 1X PBS. Vortex briefly.
  d. Add 40 μL of a 1N HCl solution and measure pH using pH-indicator strips.
  e. If necessary, adjust the pH of the solution by adding small volumes of the 1N HCl solution.
    ***Note:*** Add 1N HCl until the pH indicator strip color matches that of pH of ∼5 in the pH color scale.
    **CRITICAL:** If a pH lower than 5 is reached, proceed to the fibrillation step anyways. Do not correct the acidity of the solution with NaOH. We have observed a dramatically reduced fibrillation yield when NaOH is added to a fibrillation mixture.
  f. Incubate for 12-18h with constant rotation at 37 °C in a convection oven.
  g. After incubation, centrifuge the mix at 21,000 × *g*. for 10 min. Carefully remove the fluorescent supernatant without disturbing the pellet.
  h. Resuspend pellet in 1 mL of 1X PBS and spin again.
  i. Repeat steps g–h above until the supernatant appears clear.
    **CRITICAL:** The reaction mixture will contain unreacted soluble Aβ_42_ peptides that need to be completely eliminated by several centrifugation steps. This will ensure that only fibrillar Aβ_42_ species (and not soluble ones) are being linked to the streptavidin surfaces (see below).
30. Resuspend the washed pellet in 2 mL of 1X PBS. Sonicate for 10 min in a water bath sonicator.
31. After sonication, triturate the aggregates.
  a. Split the volume into two microcentrifuge tubes (1 mL each).
  b. Triturate by energically loading/unloading the solutions into/from a 0.5 mL insulin syringe with a 28-gauge needle. Repeat loading/unloading 20 times.
  c. Repeat the sonication and trituration cycle once more. This will ensure homogeneous aggregate size.
  d. Combine the two triturated aliquots (2 mL, final 127 μM fAβ_42_ species in solution) and store at 4°C.

**CRITICAL:** When retrieving the aggregate solution from storage at 4°C, spin it down and discard the supernatant. Then resuspend the pellet and re sonicate and triturate it prior to labeling new coverslips.

***Note:*** fAβ_42_ in Alzheimer’s senile plaques has been shown to adopt β-sheet structures,^22^ and we typically verify that our synthetic aggregates possess equivalent β-sheet features using electron microscopy and circular dichroism.^23^ For a detailed protocol on how to conduct these quality-control assessments, please refer to Jacquet et al.^1^

### Immobilization of small fAβ_42_ aggregates labeled with fluorescein and biotin on streptavidin-coated glass coverslips

**Timing: 24 h**

32. Prepare 35-mm dishes with attached streptavidin-coated coverslips (Nanocs, pn# CS-SV-5).
  a. Punch a 7-mm hole on a 35-mm plastic dish.
  b. Prepare a mixture of paraffin and petroleum jelly and warm it up at 37°C.
  c. Apply a thin layer of petroleum – paraffin mixture on the exterior bottom of the plastic dish and attach the coverslip to it, over the punched aperture.
    ***Note:*** The petroleum–jelly mixture will form a watertight seal on the plastic.
    **CRITICAL:** Do not reheat / reuse the mixture more than three times.
  d. Place the dish with the attached coverslip upside-down near a heater and allow the petroleum - paraffin to melt for 5-10 seconds.
  e. Allow the sealed coverslips to dry at 22°C–25°C for 30 min followed by sterilization by UV light exposure for 20 min. Store in the dark.
    ***Note:*** The center region of the sealed coverslips, underneath the punched holes, will be exposed and allow for direct incubation with water solutions.
    ***Alternatives:*** Nanocs coverslips can also be sealed on the bottom of individual wells in standard 6-well plates (VWR/Corning, pn# 351146) (Figure 3). Holes can be punched in each well using a puncher, or by melting the plastic using a hot soldering tip in contact with the plastic surface, applied on a circular fashion, to melt the plastic away. Residual plastic and indentations arising from the melting can be removed using grit sandpaper or a fine file. This adaptation allows for semi-high-throughput imaging capabilities.
    ***Alternatives:*** 96-well plates coated with streptavidin (pn# Streptawell, Sigma) can also be used. The plates can be labeled with aggregates by following the exact same procedure described above, applied to individual wells.

**Figure 3:**
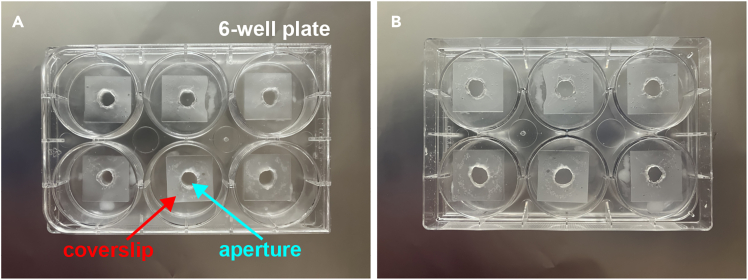
Streptavidin-coated Nanocs coverslips sealed on the bottoms of individual wells in standard 6-well plates (A) Circular apertures were opened on each well by melting the plastic on a circular fashion using a hot soldering tip. Coverslips were thereafter sealed over the apertures using a mixture of paraffin and petroleum jelly as described in step 32. (B) Opposite side of the plate, with sealed coverslips.
33. Immobilize fluorescein-biotin- fAβ_42_ aggregates on the streptavidin-coated coverslips.
  a. Reconstitute the sealed Nanocs coverslips by incubation with 80 μL of 1X PBS in a humidified cell incubator at 37°C for 30 min.
    ***Note:*** Add the 1X PBS solution inside the punched hole with exposed coverslip area.
  b. Remove the 1X PBS from the dish and add 80-100 μL of a 127μM fAβ_42_ sonicated aggregate solution (see ‘preparation of small fAβ42 aggregates labeled with fluorescein and biotin for surface immobilization*’* section above) into each punched hole.
  c. Incubate the dishes for 12-18h at 37°C in a humidified incubator.
    ***Note:*** 3 dishes should be labeled for each experimental condition being tested.
  d. After incubation, carefully recover the aggregate volumes from each dish, consolidate into a microcentrifuge tube and store at 4 ºC for eventual reuse.
    ***Note:*** Aggregate solutions can be reused up to 3 times for additional labeling.
  e. Carefully wash labeled dishes 3X with 2 mL of sterile 1X PBS.
  f. Add 80-100 μL of a 0.5 mg/mL (2 mM) biotin solution (prepared in 1X PBS) into each punched hole.
    ***Note:*** This will block unreacted surface streptavidin.
  g. Cover the dishes with aluminum foil and shake gently for 6 hours between 20 and 22 ºC.
  h. Wash dishes 3X with 2 mL of sterile 1X PBS.
    **CRITICAL:** Do not allow the dishes (or plate wells) to dry. Leave 2 mL of 1X PBS inside each dish, or 80-100 uL inside each well, during manipulation and storage steps.
  i. Gently scratch the center of each coverslip with a sterile glass pipette.
    **CRITICAL:** This will create landmarks that can be identified and used to orient the dishes and ensure that the same regions are being acquired for all timepoints.
    ***Note:*** If using a 6-well plate with sealed coverslips, or a 96-well Streptawell plate, imprint a landmark at the center of a control well (which will not be receiving any seeded microglial cells). This landmarked well will be used to adjust the orientation of the plate prior to imaging (see Figure 4, Figure 5 and ‘Monitoring the degradation of surface-immobilized small fAβ42 aggregates by digestive exophagy’ section, step 26c).

**Figure 4:**
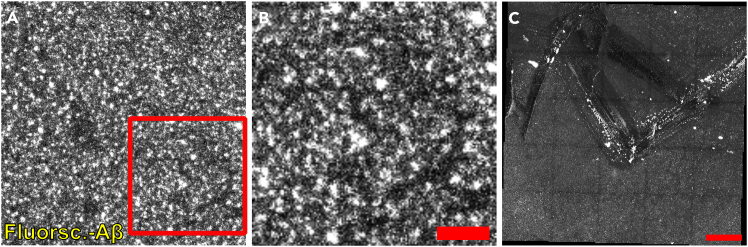
**Immobilized** fAβ_42_-fluorescein-biotin aggregates on streptavidin-coated glass coverslips. (A and B) Representative image of immobilized small aggregates (A) and inset of the area delineated by a red square in A. Scale bar: 50 μm. (C) Mark imprinted on a control chamber using a sterile glass pipette prior to seeding microglial cells, rendering a reference landmark that will be used to orient dishes or plates prior to each imaging session. This will ensure that the same regions are consistently being acquired throughout all timepoints. Scale bar: 200 μm.

**Figure 5:**
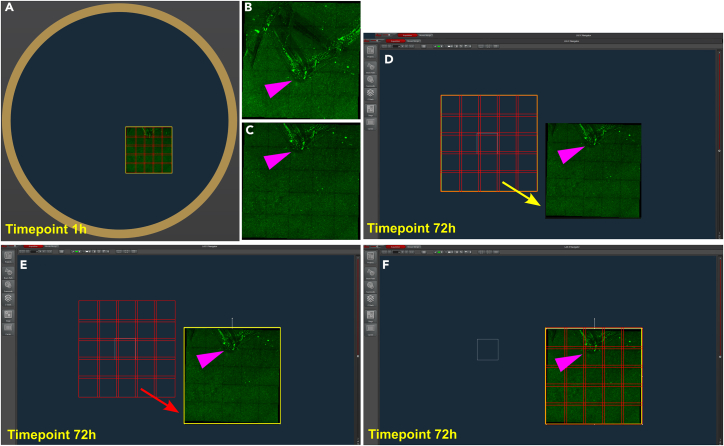
Localization of imprinted landmarks and orientation of coverslip dishes or plates during subsequent imaging sessions (A–C) Leica software imaging interface showing a diagram of a chamber and a 5x5 grid delineated on it. The resulting acquired tile image shows an easily identifiable mark previously imprinted on the chamber surface using a sterile glass pipette (B). The grid was slightly displaced downwards to capture the tip of the imprinted mark (purple arrowhead) on the upper middle region of the field (C). The grid was saved as a native region file in the imaging software. (D–F) Imprinted landmark located on the same chamber 72h after the initial imaging timepoint, matching the original landmark snapshot (shown in C). The saved 5x5 grid was loaded on the chamber but its new location did not exactly match that of the imprinted landmark (D). To correct for the spatial divergence, the loaded grid was moved to match the original landmark position (yellow square in E) and the grid automatically moved with the region (red grid in F). When imaging streptavidin-coated Nanocs glass coverslips, each individual dish must be reoriented to match the time 1h localization. If imaging a custom-made 6-well plate or a commercial 96-well Streptawell plate, all regions in all wells can be selected together with the reference 5x5 grid loaded on a control well, and will undergo the same spatial displacement as the control grid when matching it to the reference landmark snapshot.
  j. Verify aggregate immobilization and size uniformity by widefield fluorescence microscopy (see Figure 4).
  k. Verify that the landmarks on each coverslip are visible and easily identifiable (see Figure 4).
  l. Store the dishes (or plates) containing 1X PBS (to prevent drying) at 4 °C until use

## Key resources table

**Table:** 

REAGENT or RESOURCE	SOURCE	IDENTIFIER
**Chemicals, peptides and recombinant proteins**		

Aβ_42_ peptide (human) sodium salt	Anaspec	AS-60883
Aβ_42_ peptide, (human) biotinylated	Anaspec	AS-24640
Biotin	Sigma-Aldrich	B4501
Bovine Serum Albumin (BSA)	Sigma-Aldrich	A2153
DMEM cell culture medium	Corning VWR	45000–316
DMEM medium without phenol red	Gibco	31053028
DMSO	Sigma-Aldrich	34869
D-(+)-glucose powder	Sigma-Aldrich	G7021
DNAse I	Worthington Biochemical	LS002139
Fetal Bovine Serum	Thermo Fisher Scientific	10437028
Fluorescein-biotin-dextran, 10 KDa	Thermo Fisher Scientific	D7178
Forceps, fine tip	Fine Science Tools	11252–00
Forceps, curved tip	Lawton	09–0019
Hydrochloric acid, 36–38%	Fisher Scientific	02-03-053
Hoechst 33342	VWR	15547
L-glutamine	Thermo Fisher Scientific	25030081
Micro spatula	Fisher Scientific	21-401-15
Magnesium sulfate heptahydrate	Sigma-Aldrich	M2773
NHS-PEG4-biotin	VectorLabs	QBD-10200-50
NHS-Alexa 647	Thermo Fisher Scientific	A20106
NHS-Fluorescein (5/6-carboxyfluorescein)	ThermoFisher Scientific	46410
Operating Scissors	ROBOZ	RS-6808
pH-indicator paper strips	VWR	BDH35309.606
Paraformaldehyde	Electron Microscopy Sciences	19210
poly-d-lysine	Sigma-Aldrich	P1149
Potassium chloride	Sigma-Aldrich	P5405
Potassium phosphate monobasic	Sigma-Aldrich	P5655
Penicillin/streptomycin	Thermo Fisher Scientific	15140163
Sodium bicarbonate	Sigma-Aldrich	S-5761
Sodium chloride	Sigma-Aldrich	S9625
Sodium phosphate dibasic heptahydrate	Sigma-Aldrich	S9390
Sodium phosphate monobasic anhydrous	Sigma-Aldrich	S8282
Sodium tetraborate decahydrate	Sigma-Aldrich	S9640
Streptavidin	Thermo Fisher Scientific	21125
Streptavidin-coated ∼6-μm ø Nile red beads	Spherotech	SVFP-6056-5
Streptavidin-coated ∼6-μm ø purple beads	Spherotech	SVFP-6062-5
Triton X-100	Sigma-Aldrich	X100
TRIZMA base	Sigma-Aldrich	T1593
Trypsin	Worthington Biochemical	LS003707
Tweezers, fine tip	Electron Microscopy Sciences	78319-4A

**Deposited data**		

Example raw data sets and analyzed raw data	This paper	WCM Institutional Data Repository for Research (WIDRR)

**Experimental models: cell lines**		

Primary microglial cells from mouse neonates (postnatal days 2–3)	Laboratory of Dr. Frederick Maxfield	N/A

**Experimental models: organisms/strains**		

C57BL/6J wild-type mice (2–6-month-old, sex not considered)	Jackson Laboratories	000664

**Software and algorithms**		

MetaMorph v.6.7.1 for Windows	Molecular Devices	www.moleculardevices.com
GraphPad Prism v.10.3.1 for Windows	GraphPad Software	www.graphpad.com
FIJI (ImageJ v.1.54f) for Windows	Schindelin et *al.* Nat Methods (2012)	https://imagej.net/software/fiji

**Other**		

500 mL bottle filter tops	VWR	73520–992
96-well plates with glass-like polymer bottom	Cellvis	P96-1.5P
Confocal microscope	Leica	Stellaris 8
6-well plates, non-TC treated	VWR/Corning	351146
pH-meter	Thermo Fisher Scientific	Orion Star A211
Spectrophotometer/plate reader	Molecular Devices	M3
T75 tissue culture-treated flask	Corning	353136
Orbital shaker rotator	Barnstead	Lab-Line 2314
Convection oven	Fisher Scientific	Isotemp 151030513
Heater blower	Sage Instruments	279
Shaker rotisserie	Barnstead	Thermolyne C400110
Confocal microscope	Leica	Stellaris
Brightfield fluorescence microscope	Zeiss	Axiovert 7
Water-jacketed CO2 cell incubator	Precision Scientific	Napco 6301-0
15-mL centrifuge conical tubes	labForce	1158R11
50-mL centrifuge conical tubes	labForce	1158R09
Benchtop centrifuge	Eppendorf	5702
Benchtop centrifuge	Thermo Scientific	Heraeus Multifuge X3R
Petri (tissue culture) dishes	Avantor – VWR	734–2796
Water bath incubator	VWR	89032–220
Glass Pasteur pipettes	VWR	14673–043
Dialysis cassette, 3.5 KDa cutoff	Thermo Fisher Scientific	66330
Insulin syringes, 28-G ½, 0.5 mL	BD	329461
Water bath sonicator	Avantor	97043–936
Weller Soldering Station H-10799	Weller/ULINE	H-10799

## Materials and equipment

**Table:** 10X PBS

Reagent	Final concentration	Amount
NaCl	80 mg/mL	320g
HNa_2_O_4_P	26.75 mg/mL	107g
KCl	2 mg/mL	8g
H_2_KO_4_P	2.4 mg/mL	9.6g
Distilled H_2_O	N/A	4L
**Total**	**N/A**	**4L**

Store at −4 °C for up to 3 months.

**CRITICAL:** All substances are irritants to skin, eyes and respiratory system.

**Table:** Growth Medium for Microglia (with or without phenol red)

Reagent	Final concentration	Amount
DMEM (+/− phenol red)	N/A	450 mL
L-glutamine	5%	5 mL
Pen/Strep	5%	5 mL
Fetal Bovine Serum (BSA)	10%	50 mL
**Total**	**N/A**	**500 mL**

Store at 2–8 °C for up to three weeks.

**Table:** CMF-PBS buffer

Reagent	Final concentration	Amount
10X PBS	10%	100mL
D-(+)-glucose	2 mg/mL	2g
NaHCO_3_	0.04 mg/mL	0.04g
Distilled H_2_O	N/A	900 mL
**Total**	**N/A**	**1L**

Store at 2–8 °C for up to three weeks.

**Table:** Trypsin Solution

Reagent	Final concentration	Amount
Trypsin	10 mg/mL	600 mg
DNAse	1 mg/mL	60 mg
MgSO_4_	1.6 mg/mL	96mg
CMF-PBS	N/A	60 mL
**Total**	**N/A**	**60 mL**

Aliquot at 3 mL in 15 mL centrifuge conical tubes. Store at −20 °C for up to 6 months.

**CRITICAL:** Both Trypsin and DNAse are an irritant to skin, eyes, and respiratory system.

**Table:** DNAse Solution

Reagent	Final concentration	Amount
DNAse	0.06 mg/mL	0.06g
Growth Medium for Microglia	N/A	120 mL
**Total**	**N/A**	**120 mL**

Aliquot at 3 mL in 15 mL centrifuge conical tubes. Store at −20 °C for up to six months.

**CRITICAL:** DNAse is an irritant to skin, eyes, and respiratory system

## Step-by-step method details

### Measuring lysosomal exocytosis of dextrans toward fAβ_42_ aggregates

**Timing: 2–3 days (for steps 1–11)**

**Timing: 2–3 h (for steps 12–14)**

**Timing: 6 h (for steps 15–21)**

1. Coat the wells of a glass-like polymer 96-well plate with poly-d-lysine.
  a. Add 100 µL of a 0.1 mg/mL poly-d-lysine solution (prepared in 1X PBS) to each well. Incubate 1h at 37 ºC.
  b. Wash 3X in sterile 1X PBS.
  c. Store in 1X PBS at 4°C if not immediately used.
2. Follow ‘Primary Murine Microglial Cell Extraction’ protocol for harvesting microglial cells from cultured flasks.
3. Seed coated wells with 20,000 microglial cells per well in complete DMEM medium without phenol red.
4. Allow cells to settle for 1–2 h inside a humidified cell incubator at 37 °C and 5% CO_2_.
5. Load microglial LE/Ly compartments with fluorescein dextrans.
  a. Carefully remove DMEM media from wells and add 80 μL of a 10 KDa fluorescein-biotin-dextran solution at 1 mg/mL (in complete DMEM medium).
  b. Return plate to 37 °C and 5% CO_2_ and incubate for 12h.
  c. After incubating for 12h, remove medium and wash wells 2X with fresh DMEM very gently.
  d. Add 100 μL of fresh DMEM medium to each well.
  e. Chase the cells for 4 h in the incubator.
    ***Note:*** While dextrans are added 1–2 h after seeding, we allow microglial cells to incubate with the polymers for 12 h, followed by a chase period the next day in fresh medium (see below). Therefore, microglia have **more than 24 h of recovery** before imaging or interaction with extracellular aggregates. After this recovery period, cells return to their expected well-adhered, amoeboid morphologies, indicating that the cells are fully recovered before the assay begins.
  f. After the chase, add 2–5 μL of streptavidin-Alexa-647-fAβ_42_ aggregates.
    ***Note:*** The stock concentration of fAβ_42_ aggregates should be 170 μM fAβ_42_, for a final concentration of ∼3–8 μM in each well.
6. Add 2–5 μL of an undiluted, commercial 6 μm-diameter streptavidin-coated fluorescent purple bead stock solution directly into each well.

***Note:*** Beads will serve as internal control for lysosomal exocytosis specificity toward Aβ aggregates.

7. Incubate the cells with aggregates and beads in the incubator at 37 °C in 5% CO_2_ for 90 min.
  a. After incubation, remove medium from each well and wash cells 1X with fresh DMEM very gently.
8. Block wells with excess biotin and bovine serum albumin (BSA).
  a. Remove DMEM media from wells and add 100 μL of a 1 mM biotin containing 50 mg/mL BSA (in DMEM medium).
  b. Incubate cells in blocking medium at 37 °C in 5% CO_2_ for 10 min.
  c. Remove blocking medium and wash 1X in fresh DMEM very gently.

***Note:*** Incubation in biotin and BSA will block unoccupied streptavidin (not bound to biotin-fluorescein-dextran) on fAβ_42_ aggregates and reduce aggregate autofluorescence.

**CRITICAL:** All washing steps should be carried out very gently to avoid lifting the cells, aggregates or beads.

9. Fix cells in paraformaldehyde.
  a. Remove DMEM media from each well and add a 100 μL of a 1% paraformaldehyde solution (in 1X PBS).
  b. Fix for 15 min between 20 and 22 °C.
  c. Carefully remove fixative and wash wells 2X with 1X PBS very gently.
10. Permeabilize cells.
  a. Carefully remove 1X PBS from wells and add 100 μL of a solution containing 1% (v/v) Triton X-100, 1 mM biotin and 50 mg/mL BSA (in 1X PBS).
  b. Incubate in permeabilization solution for 10 min between 20 and 22 °C.
  c. Carefully remove permeabilization solution and wash 1X in 1X PBS.

***Note:*** This step removes intracellular fluorescein-dextran without affecting fluorescein-dextran deposited on the aggregates.

11. Stain microglial cell nuclei for visualization.
  a. Remove 1X PBS from wells and add 100 μL of a 1 μg/mL Hoechst 33342 solution in 1X PBS.
  b. Incubate for 10 min between 20 and 22 °C.
  c. Carefully remove Hoechst solution and wash 2X in 1x PBS.
  d. Store the plate at 4°C until imaged by confocal microscopy.

**Pause point:** Take a break before imaging the plate.

12. Acquire fluorescence images using confocal microscopy.
  ***Note:*** In our experiments, we used a Stellaris confocal microscope (Leica).
  a. Select a 20X air objective.
    ***Note:*** Using an air objective will allow for switching between wells without losing focal plane.
  b. Adjust laser lines and detectors.
    ***Note:*** Hoechst (cell nucleus) should be excited with a 405 nm solid-state laser whereas fluorescein (biotin-dextran), purple dye (streptavidin-coated beads) and Alexa 647 (fAβ_42_ aggregates) should be excited with a solid-state white laser (or equivalent) adjusted to 495 nm, 579 nm or 653 nm, respectively.
    ***Note:*** Fluorescence should be detected using high-sensitivity Leica HyD detectors (or equivalent) with spectral window adjusted to collect light between 405–490 nm (Hoechst); 500–550 nm (fluorescein), 589–650 (purple beads) and 663–750 nm (Alexa 647). Acquire the various channels sequentially using line scanning mode.
  c. Adjust pinhole to 1 Airy unit.
    **CRITICAL:** Make sure to not overexpose/saturate fluorescence.
  d. Acquire 8–12 areas per well showing microglial cells contacting both fAβ_42_ aggregates and beads.
    **CRITICAL:** Acquire stacks of images for each field. Make sure to acquire the totality of the fluorescence in that field. Images should be separated by 1 μm distance in the vertical axis.
    **CRITICAL:** Acquire 12-bit stacks of images. Images with lower bit depth might become problematic during image quantification.
  e. Save all acquired stacks in the confocal imaging software native format.
13. After imaging, return the plate to 4 °C storage.
14. Import native image files from confocal native software formats into files that can be analyzed in Metamorph.
  a. Open confocal native files with FIJI Bio-Formats plugin.
  b. Save all image files in TIF format.
15. Quantify fluorescein-biotin-dextran signal colocalized with fAβ_42_ aggregates and purple beads.
  ***Note:*** The step-by-step procedure below describes the necessary steps to quantify fluorescein integrated intensity colocalized on aggregates and beads, in one TIF image file, using any digital image analysis software. For this protocol, we have used Metamorph software.
  ***Note:*** We have prepared an automated journal allowing for batch processing of all image files corresponding to a complete experiment. The automated journal and associated sub journals, threshold files and taskbar file can be found in the Supplementary Materials section, under a folder named ‘Lysosomal_exocytosis’.
  **CRITICAL:** In order to use the journal in your computer, copy the containing folder to your computer drive. Make sure to save the folder to the C drive directly ('C∖Journals' folder).
  a. Open Metamorph. Load the ‘LY_exocytosis_taskbar’.
  b. Open an image stack to be quantified.
  c. Correct every plane in the stack for background fluorescence by removing the 5^th^ signal percentile.
  d. Split the stack into ‘Blue’ (Hoechst), ‘Green’ (fluorescein), ‘Red’ (purple beads) and ‘Far red’ (fAβ_42_-Alexa 647) channels.
    ***Note:*** To do that on Metamorph, run the sub journal *‘Stack separator (4CH)*’ in the taskbar.
  e. Select the Red channel and set a threshold to include the fluorescence for purple beads.
  f. Repeat the same operation for the Far red channel to select the fluorescence for fAβ_42_ aggregates.
    ***Note:*** If using Metamorph, save the thresholds as ‘*Red.GTH*’ and ‘*Far red.GTH*’ in the ‘Lysosomal_exocytosis’ folder.
  g. Apply the thresholds above to the Red and Far red channels and generate binary masks.
  h. Apply masks, separately, to the Green (fluorescein) channel.
    ***Note:*** This will allow for filtering and selection of fluorescein signal associated with beads or fAβ_42_ aggregates only.
    ***Note:*** If using Metamorph, use the function ‘Logical AND’.
  i. Select the masked Green channel and set a threshold to include the fluorescein signal corresponding to exocytosed dextran toward fAβ_42_ aggregates or beads.
    ***Note:*** The selected signal should be clearly identifiable on, or around, the aggregates, and its intensity should be significantly above any green background. Examples of clearly exocytosed fluorescein-dextran deposited on fAβ_42_ aggregates and beads are discussed in the Expected Outcomes section.
    **CRITICAL:** The same signal threshold selection should be applied consistently throughout all image files in a given experiment.
    ***Note:*** If using Metamorph, save the threshold as ‘*Green.GTH*’ in the ‘Lysosomal_exocytosis’ folder.
  j. Measure the thresholded fluorescein signal deposited over beads or fAβ_42_ aggregates, and log the integrated intensity measurements on a spreadsheet.
    ***Note:*** If using Metamorph, quantify a few image files by running the automated journal ‘*LY exocytosis (4CH) per-plane*’ journal in the taskbar. When executed, the journal will automatedly perform the operations described above, and fluorescein integrated intensities will be logged for each image file analyzed. Specifically, the journal will:
    i. Open an Excel spreadsheet where fluorescence intensity measurements will be logged.
    ii. Log integrated intensity and thresholded area information for the ‘Red’ and ‘Far red’ channels, corresponding to purple beads and fAβ_42_-Alexa 647 aggregates, for each plane in the stack.
    iii. Log integrated fluorescein intensity colocalized with purple beads as ‘Filtered_exo_beads’ or with fAβ_42_-Alexa 647 aggregates as ‘Filtered_exo_plaques’, for each plane in the stack.
      ***Note:*** Fluorescein intensity colocalized with aggregates and beads is a direct measurement of lysosomal exocytosis toward these objects.
  k. Quantify a few image files to verify accuracy.
    ***Note:*** The fluorescein integrated intensity values being logged after quantification should match your visual impression of lysosomal exocytosis towards beads or aggregates in the images.
    ***Note:*** If using Metamorph, run the journal ‘*Close_all*’ in the journal taskbar to close all image windows open in Metamorph after you completed a specific step. This will save substantial time (compared with closing each image one by one).
  l. Finally, quantify all files in your experiment using batch quantification.
    ***Note:*** If using Metamorph, go to **Journal>Loop>Loop for all images in a directory.** Select the location of your image files, and select the journal ‘*Lysosome_exocytosis_4CH_per-plane*’. Run the loop. All integrated intensities for all stacks and channels will be logged on the Excel spreadsheet.
  m. Quantify the number of beads in each image stack using a fluorescence-based cell counter module available on your image quantification software.
    ***Note:*** If using Metamorph, run the journal ‘*Count beads (4CH)*’ in the taskbar. The journal applies the module ‘Count Nuclei’ on Metamorph adjusted to detect nuclei with an approximate min and max width of 10 and 500 μm, respectively, and an intensity above local background of 100 gray levels.
    ***Note:*** For batch bead counting using Metamorph, go to **Journal>Loop>Loop for all images in a directory.** Select the location of your image files, and select the journal ‘*4CH-SUM_count-beads*’. Run the loop. An Excel spreadsheet will open automatically and all bead counts per image file will be logged.
16. Quantify cell nuclei in each image stack.

***Note:*** You can do that either manually by visual inspection of the Hoechst staining channel or using a nuclei quantification module on your analysis software.

***Note:*** If using Metamorph, use the module ‘Count nuclei’. However, manual quantification is better, since fAβ_42_ aggregates usually exhibit some autofluorescence in the blue channel, which hampers automated nuclei quantification using Hoechst.

17. Once all quantifications are complete, sum the fluorescein integrated intensities corresponding to beads or aggregates for each plane in a stack.

***Note:*** If you quantified and logged the data on an Excel spreadsheet using Metamorph, you will get two logged columns; one for ‘Filtered_exo_beads’ and another for ‘Filtered_exo_plaques’. These values indicate the total fluorescein signal colocalized with purple beads or fAβ_42_ aggregates in the field, respectively.

18. Normalize fluorescein integrated intensity colocalized with purple beads by the number of beads in the image.
19. Normalize fluorescein integrated intensity colocalized with fAβ_42_ aggregates by the number of cells (Hoechst nuclei) in the image.
20. Plot data graphically.
  a. Normalize all integrated intensities logged in your Excel spreadsheet for each field to the ‘purple bead’ condition.
    ***Note:*** This will give an idea of how much fluorescein is being exocytosed towards fAβ_42_ aggregates relative to control purple beads.
  b. Using GraphPad Prism, plot normalized fluorescein integrated intensity toward purple beads or fAβ_42_ aggregates.
  c. Calculate descriptive statistics.
21. Assess statistical differences between experimental conditions.
  a. Open a new spreadsheet on GraphPad Prism and copy all your data points in the same column.
  b. Test the normality assumption of your data by running Shapiro-Wilk and Kolmogorov-Smirnov tests on all data points.
    i. If the data follow a normal distribution, compare the means of the two conditions (fluorescein deposition on purple beads vs. fAβ_42_ aggregates) using the unpaired two-tailed Student’s t-test.
    ii. If the variances of the two groups are significantly different, use the unpaired two-tailed unpaired Student’s t-test with Welch’s correction.
    iii. If the data does not follow a normal distribution, compare the means of the two conditions above using a non-parametric test (Mann-Whitney or Wilcoxon rank-sum test).

***Note:*** A sample data set corresponding to a full lysosomal exocytosis experiment has been stored at Weill Cornell Institutional Data Repository for Research (WIDRR) and will be made readily available upon request. The data set includes 15 regions imaged as described above and allows for practice and repetition of the approach described above. Once satisfactorily analyzed, results will show a 40-fold increase in lysosomal exocytosis of 10 KDa fluorescein-dextrans toward fAβ_42_-Alexa 647 aggregates relative to streptavidin-coated beads. An Excel spreadsheet with a complete quantification of the images is also included in the data set.

### Monitoring the degradation of surface-immobilized small fAβ_42_ aggregates by digestive exophagy

**Timing: 3–9 days (for steps 22–25)**

**Timing: 2–3 h per time point (for steps 26–29)**

**Timing: 6 h (for steps 30–35)**

22. Bring coverslip dishes (or plates) with immobilized fAβ_42_-fluorescein-biotin aggregates from 4°C storage to 37°C.
23. Seed microglial cells at 20,000 cells/well. See ‘primary murine microglial cell extraction’ section.
24. Transfer the dishes or plate to a confocal imaging chamber.

**CRITICAL:** Pre warm the confocal incubation chamber until temperature equilibrates to 37 °C prior to transferring the dishes or plates.

25. Allow for re equilibration at 37 °C with 5% CO_2_ in the confocal chamber for 30 min.
26. Acquire fluorescein images using confocal microscopy.
  ***Note:*** In our experiments, we used a Stellaris confocal microscope (Leica).
  a. Select a 20X air objective.
    ***Note:*** Using an air objective will allow for switching between wells without losing focal plane.
  b. Adjust laser lines and detectors.
    ***Note:*** Fluorescein (immobilized fAβ_42_ aggregates) should be excited with a solid-state white laser (or equivalent) adjusted to 495 nm.
    ***Note:*** Fluorescein signal should be detected using high-sensitivity Leica HyD detectors (or equivalent) with spectral window adjusted to collect light between 500–550 nm.
    **CRITICAL:** Make sure to acquire the transmitted light images. These will be used to mask out intracellular fluorescein signal (see below).
  c. Find the landmark(s) previously imprinted on each coverslip dish (see ‘immobilization of small fAβ42 aggregates labeled with fluorescein and biotin on streptavidin-coated glass coverslips’ **step 33i**.
  d. Take a snapshot of the landmarks and save the images.
    ***Note:*** These areas will be used to verify the correct orientation of the dishes or plates on the confocal imaging chamber at different imaging time points,
  e. Select and apply a 5×5 field imaging grid containing the visible landmark on each coverslip dish.
  f. Apply the 5×5 field to each chamber and save the areas on your imaging settings.
    **CRITICAL:** These grids will be reloaded at the beginning of all subsequent imaging sessions and re acquired.
    ***Note:*** if imaging a 96-well Streptawell plate, locate the landmark imprinted on the control (no microglia well) and take a snapshot as described in steps 26c to 26f above.
  g. Adjust the pinhole to render a ∼2.5–2.7 μm-thick optical slice.
    ***Note:*** To achieve that for a 20X air objective with NA of 0.75 and acquiring fluorescein green light (520 nm), the pinhole will have to be set to approx. 2 Airy units.
  h. Acquire stacks of images using the 5×5 grid areas defined previously and save images.
    **CRITICAL:** Each stack should be composed of 12–13 planes, each with a separation of 1 μm in the vertical axis. Acquire 12-bit stacks of images. Images with lower bit depth might become problematic during image quantification. Finally, make sure that all your fluorescein signal is included within the stack.
  i. After all dishes have been imaged, return them to the incubator at 37 °C.
27. Re image all dishes or wells for all subsequent time points (24-48-72h and 7 days if necessary).
  a. Set each dish in the confocal imaging chamber and allow to re equilibrate at 37 °C and under CO_2_.
  b. Locate the landmarks imprinted in each coverslip.
  c. Load the previously saved 5×5 grid areas for each coverslip dish.
  d. Orient the grids to match the specific location acquired for the 1h timepoint (see Figure 5).
    ***Note:*** To guide you through this process, open the landmark snapshot(s) acquired during the 1h timepoint and ensure they match the current landmark region(s) imaged with the 5×5 grid.
    i. Re acquire all dishes and save image stacks.
    ii. Return dishes to the incubator.
    ***Note:*** if imaging custom-made 6-well plates or commercial 96-well Streptawell plates, load the 5×5 grid areas for all wells. After that, select all grids, including the grid corresponding to the control (no microglial cells) well. Displace the control grid to match the location originally established at time 1h. All other 5×5 grids will displace equally, matching their location at time 1h (see Figure 5).
    ***Note:*** This will allow for control of day-to-day imaging fluctuations.
28. Image Nile red fluorescent beads at the end of each imaging session.
  a. Add 50 μL of Nile red fluorescent beads on top of a microscopy-grade glass slide.
  b. Cover the applied bead solution with a 1-mm glass coverslip. Seal with nail polish.
    ***Note:*** Mounted bead slide should be stored at 4 °C and can be imaged for a month.
  c. Bring the bead slide into the confocal incubation chamber at 37 °C. Turn off CO_2_ supply.
  d. Allow slide to equilibrate to 37 °C for 10 min.
  e. Select and apply a 5×5 field grid to include as many beads as possible.
    **CRITICAL:** In order to image the same fields during subsequent imaging sessions, the grid must be applied on an area of the slide near a few identifiable landmarks.
  f. Save the grid in your imaging settings.
    **CRITICAL:** The same grid should be reloaded on the same slide region when re imaging the bead slide at the end of each time point imaging session. Imaging the same region on the slide (and therefore the same beads) will ensure tight control over fluctuating laser power and optical elements.
  g. Acquire a stack of images for each field in the 5×5 grid set on your slide. Save the images.
    **CRITICAL:** Acquire the beads with the exact same imaging settings and laser power output used to image immobilized fluorescein aggregates.
29. Import native image files from confocal native software formats into files that can be analyzed in Metamorph.
  a. Open confocal native files with FIJI Bio-Formats plugin.
  b. Save all image files in TIF format.
  c. Once all files have been generated, organize them into folders by corresponding condition and time point.

***Note:*** If using Metamorph to quantify the images, place a number (starting by '01') in front of each folder and subfolder name. Our Metamorph automated journal will access folders and quantify image fields in the numerical order determined by the folder numbering. This will allow for an orderly logging of integrated intensity values in the Excel log sheet opened at journal startup.

**Figure 6:**
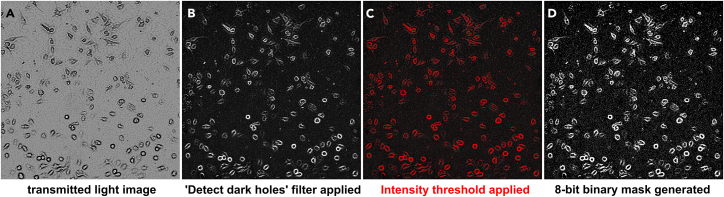
Generation of a cell mask using the transmitted light image and the ‘holes’ morphology filter on Metamorph
    vii. Open the Integrated Morphometry Analysis module on Metamorph.
    viii. Select a filter size that filters out small, pixel-size objects in the ‘dark holes’ binary mask.
    ix. Save the filter size as ‘and ‘Transmitted-light_discard-dark-holes.IMA’.
    x. Under integrated morphometry analysis, apply the selected filter size and clean the image.
    xi. Transform the filtered image into a new 8-bit binary image mask (see Figure 7).
      ***Note:*** This will eliminate from the final mask objects that are not associated with cells such as small debris, or some small aggregates that appear in the transmitted light image.

30. Quantify surface-immobilized fluorescein-fAβ_42_ signal:
  ***Note:*** The step-by-step procedure below describes the necessary steps to quantify fluorescein integrated intensity in one TIF image file, using any digital image analysis software. For this protocol, we have used Metamorph software. We have prepared an automated journal allowing for batch processing of all image files in a complete experiment. The automated journal and associated sub journals, threshold files and taskbar file can be found in the Supplementary Materials section, in a folder named ‘Degradation-assay’. Copy this folder to your computer drive (C∖Journals folder).
  a. Load an image stack to be quantified into your analysis software.
  b. Correct every plane in the stack for background fluorescence by removing the 5^th^ signal percentile.
  c. Split the transmitted image and fluorescein channels and generate a sum projection of each channel.
    ***Note:*** If using Metamorph, load the ‘Degradation-Assay_Taskbar’ taskbar. Run the sub journal ‘*2-CH separator pc correction*’.
  d. Subtract intracellular fluorescein signal.
    ***Note:*** For 24, 48, 72 h and 7-day time points: cells will have already lifted and internalized some of the surface-immobilized fluorescein-fAβ_42_ and will contain a substantial amount of intracellular fluorescein. In order to quantify signal from fluorescein-fAβ_42_ aggregates that remains immobilized **only**, we need eliminate any intracellular fluorescein signal. You can skip this step for 30 min time points (t_0_), since the cells will not show any significant intracellular fluorescein-fAβ_42_ incorporation. To remove intracellular fluorescein signal, we will use the transmitted light image to dissect cells and generate a mask (see below).
    ***Note:*** If you use Metamorph, jump to step e.
    i. Select the transmitted light image.
    ii. Segment cells by applying a threshold or using a segmenting tool.
    iii. Generate a negative binary mask.
      ***Note:*** In the binary mask, areas corresponding to cells should contain a pixel value equal to ‘0’.
    iv. Apply the mask to the fluorescein channel.
      ***Note:*** Once applied, intracellular fluorescein signal will be multiplied by '0' and eliminated from the image.
  e. Subtract intracellular fluorescein signal **using Metamorph** automated journal.
    ***Note:*** We prepared an automated journal entitled ‘*Remove-cells-using-transmitted-channel_filtered.JNL*’ that eliminates intracellular fluorescein signal by applying a negative mask generated using the transmitted light image. To use the journal, it is necessary to adjust a few settings (see below).
    i. Open the journal taskbar ‘*Degradation-Assay_Taskbar*’.
    ii. Open a degradation assay image stack.
    iii. Click on ‘2CH separator 5pc correct’ option in the taskbar.
      ***Note:*** This will split the opened stack into fluorescein and transmitted light channels, correct each image in the stack by subtracting the 5^th^ signal percentile and generate a total sum projection for each channel.
    iv. Go to *Process>Morphology Filters.* Select ‘Holes’ and ‘Detect dark holes’ and apply it to the transmitted light channel.
      ***Note:*** Cell membranes appear in a distinct contrast in the transmitted light image. Some regions of the membrane are dark, whereas others appear brighter. This operation will segment the dark contrast regions of cells in the transmitted light image.
    v. Select a threshold that includes the segmented dark contrast regions and save it as ‘*Holes_dark.GTH*’ threshold file in your journal folder.
    vi. Generate an 8-bit binary mask using the applied intensity threshold to the ‘Dark Holes’ image (see Figure 6).

**Figure 7:**
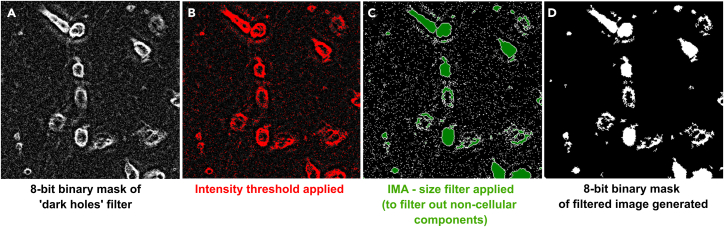
Elimination of small objects in the 8-bit binary mask Elimination of small, pixel-sized objects (A, and thresholded image in B) from the cell mask using the integrated morphometry analysis module (C and resulting image D) in Metamorph.
    xii. Go to Process>Morphology filters and dilate the image using a 6-pixel circular filter.
    xiii. Go to Process>Morphology filters and apply the ‘open-close’ function with a 6-pixel circular filter.
    xiv. Go to integrated morphometry analysis and select a filter size that filters out small, pixel-size objects in the previous ‘open-close’-filtered image.
    xv. Save the filter size as ‘and ‘Filter-open-close-holes.IMA’.
      ***Note:*** This will slightly enlarge the segmented cells and close up any unselected area within their segmented bodies (see Figures 8A and 8B).

**Figure 8:**
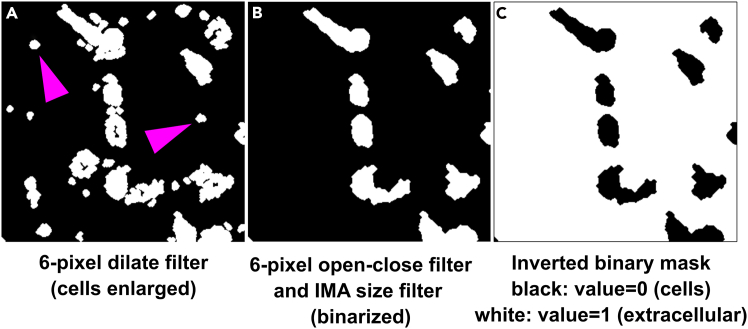
Morphology filters applied to the 8-bit binary mask for its optimization ‘Dilate’ morphology filter applied to the cell binary mask (A) followed by the use of the ‘Open-close’ morphology filter to close circular objects (D) and elimination of small extracellular debris using integrated morphometry analysis module in Metamorph (arrowheads in A and resulting image in B). The resulting mask is then inverted to yield a gray scale value of ‘0’ for pixels representing cell somas and a value of ‘1’ to the extracellular space (C).
    xvi. Invert the previously filtered image (see Figure 8C).
      ***Note:*** Regions corresponding to cells should have a grey scale value of 0, whereas all remaining area should be assigned a grey scale value of 1.
    xvii. Multiply the fluorescein channel by your finalized ‘dark holes’ mask. All regions with a grey scale value of 0 will be eliminated from the fluorescein channel (see Figure 9).

**Figure 9:**
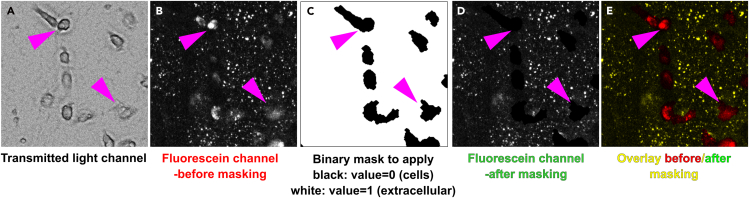
Subtraction of intracellular fluorescein signal by a binary mask generated using the transmitted light channel Microglial cells (A, arrowheads) show fluorescein signal (B, arrowheads) which can be eliminated by multiplying the fluorescein channel (B) by a binary mask (C) that eliminates cell-associated fluorescein signal (D, arrowheads). The overlay of images before (B) and after (D) intracellular signal correction shows that only intracellular fluorescein signal is being eliminated from the fluorescein channel (E, arrowheads).
    xviii. Repeat operations iv to vi using the ‘Detect light holes’ function and complete steps vii to xvii in identical manner as described above.
      ***Note:*** The workflow corresponding to ‘Detect light holes’ filter is identical to ‘Detect dark holes’ function but this time Metamorph will dissect portions of cells by segmenting their brighter contrast regions. In step v, select a threshold that selects the segmented cells and save it as ‘*Holes_light.GTH*’ threshold file in your journal folder. After that, in step ix, select a filter size using the integrated morphometry analysis module and save it as ‘*Transmitted-light_discard-light-holes.IMA*’.
      ***Note:*** The combined used of ‘Dark holes’ and ‘Light holes’ segmentation masks will yield a complete representation of the area occupied by the cells in the transmitted light image and ensure that all intracellular signal is removed from the fluorescein channel
    xix. To test your ‘dark holes’ mask settings, re open your image stack and run the journal ‘*Intracellular-corr_DARK-TEST’* journal in the taskbar.
    xx. To test your ‘light holes’ mask settings, re open your image stack and run the journal ‘*Intracellular-corr_LIGHT-TEST’* journal in the taskbar.
    xxi. Finally, To test your combined ‘dark holes’ and ‘light holes’ masks, re open your image stack and run the journal ‘*Intracellular-corr_ALL-TEST’* journal in the taskbar.
      ***Note:*** Testing the masks separately first will allow for fine tuning of settings. The subtraction for both masks from the fluorescein channel will eliminate intracellular signal from the fluorescein channel sum projection.
    xxii. Test your masking settings for a few image files, for all time points.
      ***Note:*** Testing the correction mask on different fields and time points will help ensure that fluorescein intracellular signal is consistently being eliminated.
      ***Note:*** For further verification, save total projections of the fluorescein channel before and after intracellular correction. Open them in Metamorph and generate an overlay (assign red and green colors for before and after corrections, respectively). Extracellular fluorescein-fAβ_42_ should appear yellow in your overlay, whereas eliminated intracellular fluorescein should appear red only, and colocalize with cells in the transmitted light image (see Figure 9E).
  f. Quantify fluorescein-fAβ_42_ signal in corrected images.
    i. Select a threshold to include the signal corresponding to undegraded surface-immobilized fluorescein-fAβ_42_ in a few corrected image stacks.
      ***Note:*** To do that accurately, open an image from your latest time point and select a threshold that excludes regions with visibly lifted fAβ_42_. This is easy to spot, since cells usually leave a circle of lifted fibrillar material around them.
      ***Note:*** If using Metamorph, adjust the green threshold and save it as ‘*Green.GTH*’ on your journal folder.
  g. Measure thresholded fluorescein signal in a few corrected images for all time points and log the measurements.
  h. Verify that the logged fluorescein intensity values reflect your visual impression of the remaining fluorescein signal in the images.
    ***Note:*** If using Metamorph, run the journal ‘*Q-extracellular-cells-removed*’ journal in the taskbar for a few images. When executed, the journal will perform all operations described above for the currently open image file. An Excel spreadsheet will open and fluorescein integrated intensity will be logged.
  i. Quantify all files in your experiment using batch quantification in your analysis software.
    ***Note:*** If using Metamorph, go to **Journal>Loop>Loop for all images in a directory.** Select the location of your files, and select the journal ‘*Degradation_quant_extracellular_cells-removed-filtered*’. Run the loop. All integrated intensities for all stacks will be logged on your Excel spreadsheet in the order determined by your folder name numbering.
31. Quantify average bead intensity (on glass slide).
  ***Note:*** If you use Metamorph for quantification, jump to step e.
  a. Open a bead image stack.
  b. Correct each image in the stack for background fluorescence by removing the 5^th^ signal percentile.
  c. Split the stack into Nile red beads and transmitted light image and generate the sum projection of the stacks.
  d. Quantify average bead integrated intensity per field. Repeat for each image file.
    ***Note:*** Modern confocal microscopes are equipped with very stable laser lines. Nile red bead intensities should be very similar between independent experiments, provided that the same imaging setting are used to acquire the images.
  e. If using Metamorph, open a bead image stack and run the journal ‘*2CH separator 5pc correct*’ in the taskbar.
    ***Note:*** This journal corrects each image in the stack by subtracting the 5^th^ fluorescence percentile and generate sum projections for the Nile red beads and transmitted light channels.
  f. Select an intensity threshold for the sum projection of the Nile red bead channel to include the signal from the beads.
  g. Save the threshold as ‘*bead. GTH*’ in the journal folder.
  h. Run the journal ‘*Quantify-bead-intensity’* in the taskbar for a few bead files to verify that images are being quantified correctly.
    ***Note:*** The journal will log average Nile red integrated intensities per field. Intensities for different fields should be very similar, as all beads exhibit homogeneous fluorescence.
  i. Finally, quantify all bead image stacks in your experiment in batch.
    i. Go to **Journal>Loop>Loop for all images in a directory.**
    ii. Select the location of your bead image files and the journal ‘*Quantify-bead-intensity*’ and run the loop.
    iii. Run the batch loop.
  j. Calculate the average of the column ‘average integrated intensity’ values for all fields.
32. Normalize fluorescein integrated intensities in Microsoft Excel.
  a. Normalize all integrated fluorescein intensities logged in the Excel spreadsheet to the corresponding averaged control bead intensity for that timepoint.
  b. Normalize all bead-normalized fluorescein integrated intensities for each field and time point to their corresponding time 1h integrated intensity values.

***Note:*** This will result in all ‘1 h’ timepoint fluorescein values being equal to 1. That is correct, since the same fields and wells were being imaged for all time points.

33. Plot the normalized averaged fluorescein integrated intensities for all time points using GraphPad Prism.
34. Calculate descriptive statistics.
35. Assess statistical differences between normalized fluorescein intensity means of all time points and conditions using GraphPad:
  a. Plot all data points in the same column and run the Shapiro-Wilk and Kolmogorov-Smirnov tests.
  b. If your data follows a normal distribution, compare the means of all conditions using the one-sample Student’s t-test (all conditions compared to the normalized time 0 value of ‘1’).
  c. If the data does not follow a normal distribution, compare the means of all conditions to the time 0 value of ‘1’ using the non-parametric Wilcoxon signed rank test.

***Note:*** A sample data set corresponding to a full degradation experiment has been stored at Weill Cornell Institutional Data Repository for Research (WIDRR) and will be made readily available upon request. The data corresponds to a degradation experiment in which microglia were seeded on top of surface-immobilized fluorescein- fAβ_42_ aggregates and imaged over the course of 1, 24, and 72 h. This data set allows for practice and repetition of the approach described above. Once satisfactorily analyzed, results will show a marked decrease in surface fluorescein signal over time corresponding to microglial digestive exophagy of fAβ_42_. An Excel spreadsheet with a complete quantification of the images is also included in the data set.

## Expected outcomes

Herein, we describe two approaches to quantify digestive exophagy of Alzheimer’s model fAβ_42_ aggregates by primary murine microglial cells. First, we prepare large fAβ_42_ aggregates labeled with streptavidin and Alexa 647 and quantify microglial exocytosis of LE/Ly contents towards the aggregates as a direct measure of digestive exophagy. Secondly, we quantify the degradation of small fAβ_42_ aggregates tightly immobilized on glass surfaces by biotin-streptavidin chemistry, which impedes their endocytosis or phagocytosis. Under these conditions, microglial degradation can only occur extracellularly, with secreted lysosomal enzymes, which is a second direct readout of digestive exophagy.

**Figure 10:**
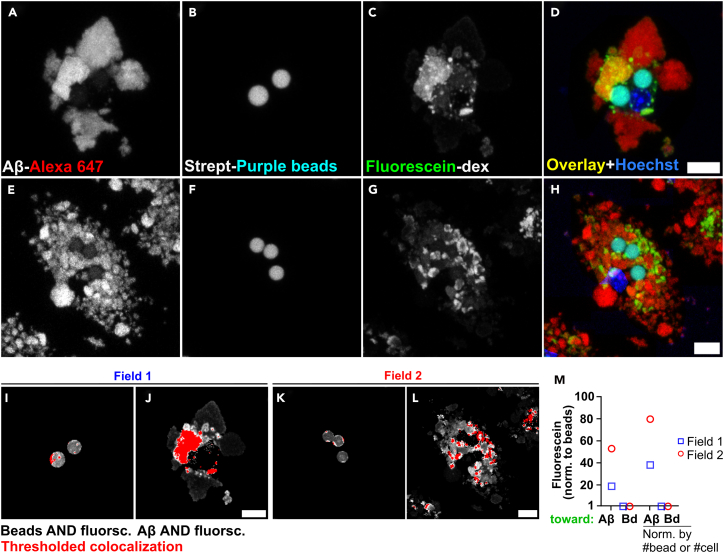
Lysosomal exocytosis of fluorescein-dextrans toward fAβ_42_ aggregates and beads (A–H) fAβ_42_-Alexa 647-streptavidin aggregates (A, E) and streptavidin-coated beads (B, F) incubated on top of microglial cells seeded on glass-like polymer wells (Hoechst staining in D and H). Cellular LE/Ly compartments were previously loaded with 10 KDa dextrans labeled with biotin and fluorescein by 12h overnight incubation. Aggregates, beads and cells were co-incubated at 37 ºC for 90 min followed by fixation in 0.5% PFA, permeabilization (in order to eliminate all remaining, non-exocytosed intracellular dextrans) and imaging to detect exocytosed dextrans toward the objects (C, G and overlays D and H). Two representative fields showing insets of individual aggregates, beads and cells are shown for illustrative purposes. In general, we acquire and quantify fields containing at least 4 large aggregates and 3-30 beads, contacted by 3-20 microglial cells (see Lysosomal Exocytosis data set). (I–M) Signal corresponding to exocytosed fluorescein dextran deposited on beads (I, K) or aggregates (J, L) was thresholded and integrated intensity was quantified for each object using digital image analysis. Thresholded fluorescein signal was normalized either to total fluorescence deposited on beads, or to the number of objects (beads or cells contacting aggregates) in the field and plotted for comparative purposes (M). Circles and squares represent these measurements for each field. Scale bars: 10 μm.

Microglial cells contacting streptavidin-coated fAβ_42_ aggregates (Figures 10A and 10E) exocytose previously internalized lysosomal dextrans towards the deposits (Figures 10C and 10G and overlays 10D and 10H). To determine the specificity of this mechanism towards fAβ_42_, control fluorescent beads also coated with streptavidin are added to the preparations Figures 10B and 10F). Following co-incubation of aggregates and beads with microglial cells, any dextrans left in LE/Ly compartments will be washed away by permeabilization with Triton, whereas dextrans exocytosed toward the streptavidin-coated objects will remain attached due to strong biotin-streptavidin interactions. Fluorescein signal deposited on beads (Figures 10I and 10K) or fAβ_42_ aggregates (Figures 10J and 10L) can be specifically segmented by digitally masking out signal not pertaining to these objects in the image. Fluorescein intensity deposited on the objects, representing lysosomal synapses, is then selected using an intensity threshold (examples of selected intensities are pseudo colored in red in Figures 10I–L) and quantified by digital image analysis. This measurement provides a quantitative assessment of lysosomal exocytosis. Generally, microglial cells exocytose dextrans towards fAβ_42_ aggregates at much higher rates relative to streptavidin-coated beads, indicating a specificity of this mechanism towards fAβ_42_ (Figures 10D, 10H and 10M).

**Figure 11:**
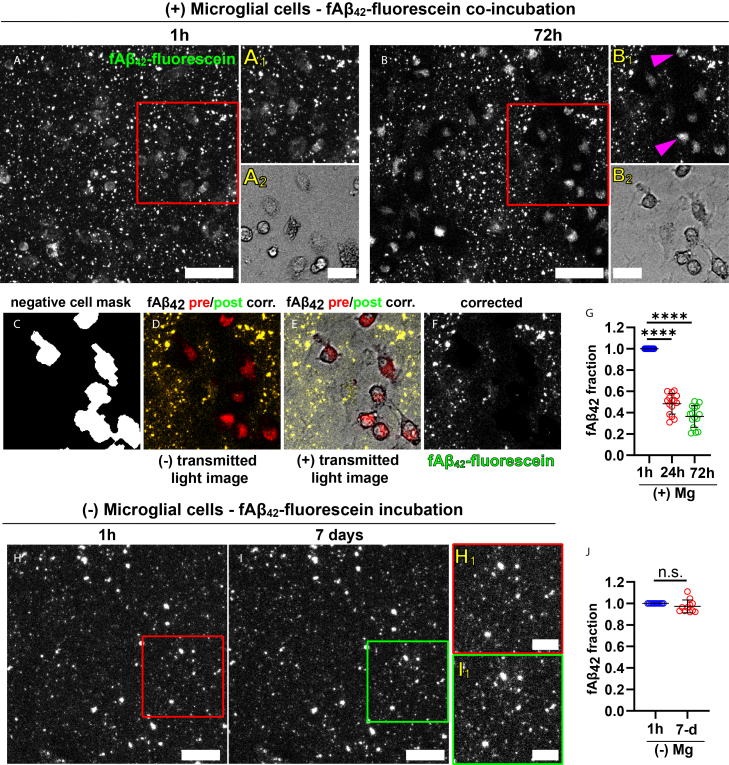
Digestive exophagy of surface-immobilized small fAβ_42_ aggregates by microglial cells (A–G) fAβ_42_-fluorescein-biotin aggregates immobilized on Nanocs glass coverslips coated with streptavidin. The coverslips were seeded with microglial cells and co-incubated for 1 h (A and insets A_1_ and A_2_ and 72 h (B and insets B_1_ and B_2_). To subtract intracellular fluorescein signal in the 72h co-incubation timepoint, a negative binary mask was generated (C) using the transmitted light images (B_2_) and applied to the fluorescein channel (B_1_). Only intracellular fluorescein signal was eliminated, whereas extracellular fAβ_42_ signal not associated with cells was preserved (overlays D and E and corrected image in F). Fluorescein was quantified for 1h, 24h and 48h timepoints in corrected images (G). The experiment was carried out once for illustrative purposes. 15 matched fields were imaged in one coverslip, for all timepoints. (H–J) Immobilized fAβ_42_-fluorescein aggregates *in absence* of microglial cells were incubated at 37 ºC and imaged at 1h (H and inset H_1_) and 7 days (I and inset I_1_) timepoints. 11 matched fields were imaged in one coverslip, for all time points. Fluorescein was quantified following the same approach as described above (J). For all plots (G and J), fluorescein signal for each field and timepoint was normalized to timepoint 1h. Circles indicate normalized fluorescein intensity measured for each field, and bars correspond to average intensity ±SD. Normalized average fluorescein intensities for timepoints 24h (images not shown) and 72h (G) or 7 days (J) were compared to the known value of ‘1’ assigned to the 1h timepoint using the one-sample Student’s t test. P-values shown as p≥0.05 (n.s.); p≤0.0001 (∗∗∗∗). Scale bars: large panels: 50 μm. Insets: 25 μm. Abbreviations: Mg: microglia.

Secondly, to demonstrate degradation of fAβ_42_ aggregates under conditions that *impede* endocytosis or phagocytosis, we attach biotinylated fAβ_42_ aggregates to streptavidin-coated glass coverslips and measure their degradation over time. Aggregates are tightly immobilized by to strong biotin-streptavidin interactions and remain attached to the surface of the coverslips despite prolonged incubation times at 37 °C. Immediately following seeding of microglial cells there is little or no fAβ_42_ degradation (Figure 11A and insets 11A_1-2_), but we observe a progressive clearance of the aggregates after 24 h (images not shown) or 72h in areas populated with cells (Figure 11B and insets 11B_1-2_*,* arrowheads). This demonstrates that the degradation occurs *extracellularly*, which is the main feature of digestive exophagy.^1^ Lifted fAβ_42_ aggregates become internalized by the cells, but microglia are not efficient at degrading fAβ_42_,^24^^,^^25^^,^^26^ which leads to intracellular fluorescein accumulation (Figure inset 11B_1_ arrowheads). In order to quantify changes in surface-immobilized fAβ_42_ *only* (indicative of digestive exophagy), intracellular fluorescein signal must be excluded from the quantification. We achieve that by generating a negative cell mask using the transmitted light image (Figure 11B_2_, and corresponding negative mask in Figure 11C) which we then apply to the fluorescein channel, effectively removing any intracellular fluorescein signal [Figures 11D and 11E, overlays showing fluorescein signal pre- (red) and post- (green) correction, with or without the overlaid transmitted light image, respectively]. Using this approach, extracellular aggregates not contacted or internalized by the cells are conserved (Figure 11F). In our experiments, we typically observe a progressive degradation of surface-immobilized fibrils in presence of microglial cells Figure 11G), whereas fluorescein in control wells devoid of cells remain unchanged over time Figures 11H–J).

## Limitations

Our methodology relies on the use of primary murine microglial cells. Most extraction protocols are known to alter microglia phenotypes and induce inflammation.^27^ Also, maintenance conditions in cell culture are dramatically different from the brain’s homeostatic environment. Nevertheless, initial cell culture studies provide invaluable guidance on what to look for in the much more complex *in vivo* studies.^1^

The *main component* of brain Alzheimer’s plaques is Aβ_42_, which is responsible for plaque β-sheet structure and aggregation.^28^ Our synthetic model aggregates are composed of aggregated Aβ_42_ only, and lack damaged cellular components, cytokines and other pro-inflammatory components present in Alzheimer’s Aβ senile plaques.^29^ Nevertheless, our primary microglial cells responded to the model aggregates and were able to exocytose lysosomal contents and slowly digest them. Microglial response might have been even more pronounced had the cells been exposed to native, multi-component Aβ senile plaques obtained from patients with AD instead.

For this STAR Protocol, we intentionally focus on the confocal microscopy workflow because it is widely accessible and allows for quantitative determination of digestive exophagy with high reproducibility. Nevertheless, alternative complimentary readouts such as supernatant dextran fluorescence quantification using spectroscopic methods, or ultrastructural characterization and quantification of digestive exophagy using electron microscopy are possible.

The immobilization of aggregates on commercially available streptavidin-coated plates might require some optimization before consistent labeling is achieved. When working with 96-well Streptawell plates, some wells might show variable aggregate immobilization densities. This is probably due to variability in streptavidin coating during manufacturing. To circumvent that, we label as many wells as possible and select those with similar immobilized aggregate densities prior to seeding with microglial cells. Sonication and trituration of the aggregates is critical, as smaller aggregates will bind to, and remain immobilized on the streptavidin surfaces easily when compared with larger aggregates. Nevertheless, we also report the preparation of custom-made 6-well plates with sealed Nanocs glass coverslips, which renders excellent aggregate immobilization and allows for semi-high-throughput capabilities.

For the degradation assay, it is also important to include a condition in which coated wells are incubated in absence of microglial cells. This will allow for monitoring of aggregate stability at 37 °C and 5% CO_2_ conditions and rule out any dislodging due to reasons other than degradation by digestive exophagy.

Finally, to measure Ly exocytosis we label LE/Ly compartments with 10 kDa dextrans. These polymers are composed of glucose subunits and are inert; they cannot be degraded by Ly enzymes and thus accumulate in Lys over time. Dextrans have been used for more than 40 years as reliable markers of LE/Lys in macrophages and microglia, and used to measure LE/Ly pH in live-cell assays. In our hands, and in previous publications from our group, incubation with 10 kDa dextrans at the concentrations used here (0.5 mg/mL or less), followed by the chase period described, does not induce microglial activation. In our digestive exophagy assays, the pH at the lysosomal synapse remains at ∼6.4^1^, indicating that microglia remain non-activated and metabolically competent while performing exocytosis toward extracellular aggregates. Similarly, for our degradation assay, fAβ_42_ is reacted with streptavidin and biotin in Aβ molar excess, resulting in moderate labeling while avoiding a high density of reactive surface groups. After surface immobilization of fAβ_42_, any remaining streptavidin binding sites are blocked with excess biotin. Thus, the microglial cells are not exposed to unbound streptavidin or biotin during the experiment. Nevertheless, if necessary, microglial inflammatory and activation status can be easily assessed by staining the cells with Iba1 or CD11b markers.

## Troubleshooting

### Problem 1: Primary murine microglia extraction

Primary murine microglial cell extraction results in low cell densities or no cells present on the flask after 10 days in the incubator.

### Potential solution

• Quality of reagents is poor. DMEM growth medium, trypsin, CMF-PBS and/or DNAse used during extraction and subsequent growing may be old and/or expired, or might have been through several freeze-thaw cycles. Prepare growth medium and CMF-PBS fresh every 1–2 months, and prepare trypsin and DNAse aliquots fresh every 5 months.
• Incorrect number of brains added to flask. Add cortical tissues from a maximum of 4–5 pup brains (8–10 cortical fragments) into each conical tube, and transfer each resulting tissue pellet to a separate.T75 flask. Plating less tissue will lead to sparse cell densities and impede growth. Plating too much cortical tissue per flask will lead to over confluence and cell activation/death.
• Incorrect amount of trypsin used. Do not add more than 1 mL of trypsinization solution per 4–5 brains (8–10 cortical fragments). Also, do not use trypsin at concentrations higher than 10 mg/mL. Using more trypsin will cause excessive trypsinization and extensive cellular damage, leading to little or no growth. Using too little trypsin will lead to insufficient trypsinization, also preventing cell growth.
• Cortical fragments left in trypsin for too long. Trypsinization of cortical tissues should be carried out swiftly to avoid excessive digestion and resulting cellular damage.
• Trypsin and DNAse not sufficiently warm when used, which dramatically reduces their proteolytic efficiency. The optimal time for an aliquot of trypsin and DNAse to warm in the water bath is 20 min.

### Problem 2: Primary murine microglia extraction

The brain is damaged when extracting it from the skull.

### Potential solution

• The skull incision was too narrow and the brain got caught under the skull and damaged during its extraction. To prevent this, ensure that skull incision is wide enough to allow sufficient room to extract the brain.

### Problem 3: Preparation of Alexa 647-streptavidin-fAβ_42_ and fluorescein-biotin-fAβ_42_ aggregates

Centrifugation of the aggregation mixture yields little to no pellet visible at the bottom of the microcentrifuge tube.

### Potential solution

• Spin down your fibrillary mix for a longer time. A mix containing a fAβ_42_concentration of 200 μM should yield a clearly visible pellet after 12h incubation at 37°C and centrifugation. If the pellet is very small or not visible, do not proceed with experiment. Instead, prepare a new aggregation mixture. Ensure that concentrations are calculated correctly, adjust the pH of the mixture between 4 and 5 using pH indicator paper strips, and verify that your incubator is equilibrated at 37 °C. An aggregation mixture with a pH below 4 is not ideal but it is still usable. Do not use NaOH to adjust the pH back to 5, as NaOH prevents Aβ_42_ aggregation.

### Problem 4: Immobilization of fluorescein-biotin-fAβ_42_ aggregates on streptavidin-coated surfaces

Following aggregate immobilization and biotin blocking, wells still show large fluorescein-fAβ_42_ aggregates and the amount of small aggregates immobilized on the surfaces is low.

### Potential solution

• Insufficient sonication and trituration of the aggregates. Dilute the aggregate preparation further by adding an extra 50% of 1X PBS volume. Re sonicate the aggregates for 10 min and repeat the trituration step. Make sure to use an insulin syringe with an attached 28-gauge needle for the trituration step. This should help break down larger aggregates and increase the number of smaller aggregates, which should bind to streptavidin-coated surfaces with higher affinity.

### Problem 5: Monitoring the degradation of surface-immobilized fluorescein-biotin-fAβ_42_ aggregates by digestive exophagy over time

Cannot locate regions initially imaged on the coverslip dishes or plates during the 1h timepoint.

### Potential solution

• A small degree of spatial variation in area location is expected when imaging the same dish or plate at different time points. However, excessive spatial mismatch should be avoided. To locate the same regions imaged during the initial 1h timepoint, locate and utilize landmarks for orientation. This can be achieved by inspecting images from previous time points and identifying landmarks such as unchanged aggregates or well surface marks (scratches) shared among all timepoints. Locate these landmarks during your initial time 1h imaging session and save the images. In subsequent imaging sessions, locate the same landmarks and calibrate the position of your plate to match the orientation from past imaging sessions.

## Resource availability

### Lead contact

Further information and requests for resources and reagents should be directed to and will be fulfilled by the lead contact, Santiago Solé-Domènech (sas2068@med.cornell.edu).

### Technical contact

Technical questions on executing this protocol should be directed to and will be answered by the technical contacts, Lucy Funes (luf404@med.cornell.edu) and Santiago Solé-Domènech (sas2068@med.cornell.edu).

### Materials availability

Further information and requests regarding ratiometric pH imaging of lysosomal synapses (protocol not included herein), or for acidic pH indicator dye (ApHID),^13^ should be directed to and will be fulfilled by the lead contact upon request.

### Data and code availability

Confocal microscopy stacks of images and all associated data analysis spreadsheets, as well as all supporting supplementary datasets for evaluation and testing of Metamorph journals are stored at Weill Cornell Institutional Data Repository for Research (WIDRR) and will be made readily available by the lead contact upon request. This paper does not report original code. Any additional information required to reanalyze the data reported in this work paper is available from the lead contact upon request.

## Acknowledgments

This work was supported by the Cure Alzheimer’s Foundation grant CAF-211540-02 and NIH grants RF1-AG078244 and R01-HL093324. S.S.-D. was supported by the Swedish Research Council International Postdoctoral Fellowship number 637-2013-503/D0050301 and the Leon Levy Foundation Fellowship in Neuroscience. The authors are grateful to Roopram Doobay for assistance in sealing glass coverslips on 6-well plates and Diane Marie Del Valle for providing advice on the preparation of the graphical abstract.

## Author contributions

Conceptualization, F.R.M., S.S.-D., and L.F.; methodology, S.S.-D. and L.F.; validation, S.S.-D. and L.F.; investigation, L.F. and S.S.-D.; resources, F.R.M. and S.S.-D.; data curation, S.S.-D. and L.F.; writing – original draft, L.F.; writing – review and editing, S.S.-D. and F.R.M.; visualization, L.F. and S.S.-D.; supervision, S.S.-D. and F.R.M.; project administration, F.R.M. and S.S.-D.; funding acquisition, F.R.M. and S.S.-D.

## Declaration of interests

The chemical synthesis and uses of the pH sensor ApHID have been included and described in a pending patent application, for which S.S.-D. and F.R.M. are co-inventors.

## Supplemental information

Data S1. Journals to quantify lysosomal exocytosis and degradation of surface-immobilized aggregates using Metamorph imaging software
